# Electrochemically Induced Structural Evolution to Generate Optimized High‐Entropy‐Alloy Electrocatalysts for Ethanol Oxidation

**DOI:** 10.1002/anie.8871821

**Published:** 2026-07-11

**Authors:** Yueh‐Chun Hsiao, Hansaem Jang, Chun‐Wei Chang, Jui‐Tai Lin, Kuan‐Fang Lee, Adrian M. Gardner, Richard J. Potter, Alex R. Neale, Laurence J. Hardwick, Kun‐Han Lin, Tung‐Han Yang, Alexander J. Cowan

**Affiliations:** ^1^ Stephenson Institute for Renewable Energy and Department of Chemistry University of Liverpool Liverpool UK; ^2^ Department of Chemical Engineering National Tsing Hua University Hsinchu Taiwan; ^3^ Low Energy Ion Scattering Facility, George Holt Building University of Liverpool Liverpool UK; ^4^ Early Career Laser Laboratory and Surface Science Research Centre University of Liverpool Liverpool UK; ^5^ Department of Materials Design and Manufacturing Engineering University of Liverpool Liverpool UK; ^6^ High Entropy Materials Center National Tsing Hua University Hsinchu Taiwan; ^7^ College of Semiconductor Research National Tsing Hua University Hsinchu Taiwan

**Keywords:** ethanol oxidation reaction, high‐entropy‐alloy nanocrystals, structural reconstruction, surface‐enhanced infrared absorption spectroscopy, x‐ray absorption spectroscopy

## Abstract

High‐entropy‐alloy (HEA) nanocrystals offer tremendous potential as next‐generation catalysts for complex electrochemical reactions. Nonetheless, there is a relative dearth of attention regarding the structural evolution of HEAs under electrochemical conditions. We herein used platinum‐group HEA nanocubes, initially enclosed by well‐defined {100} facets, as electrocatalysts for the multistep ethanol oxidation reaction (EOR). Notably, the prepared catalysts demonstrate an 8.3‐fold enhancement in specific activity during electrochemical cycling, driven by the structural evolution of catalyst facets. This transformation leads to a severely beveled cubic morphology characterized by an approximately equal distribution of {100}, {110}, and {111} facets, while preserving the compositional homogeneity and high‐entropy nature, as confirmed by high‐resolution transmission electron microscopy and synchrotron‐based x‐ray absorption spectroscopy. In situ surface‐enhanced infrared absorption spectroscopy, electrochemical stripping experiments, and computational calculations reveal that the enhanced performance originates from improved C─C bond cleavage and superior resistance to poisoning by formate intermediates (HCOO_ad_). These features promote complete oxidation of ethanol to CO_2_, a critical step for maximizing efficiency in direct alcohol fuel cells for renewable energy applications.

## Graphical abstract

Electrochemical cycling drives spontaneous facet optimization in high‐entropy Pd@PdPtIrRuRh core‐shell nanocrystals, reshaping simple cubes into severely beveled architectures with balanced {100}/{110}/{111} facets. This dynamic restructuring yields active electrocatalysts that favor complete ethanol oxidation via the C1 pathway, suppress strongly adsorbed poisons, and enable efficient direct ethanol fuel cell applications.

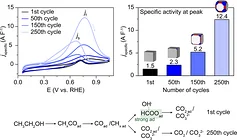

## 1 Introduction

The global move toward sustainable energy has highlighted direct ethanol fuel cells (DEFCs) as a promising clean energy option due to their high energy density and compatibility with renewable bioethanol [1, 2, 3, 4]. A key part of DEFC performance is the ethanol oxidation reaction, EOR, a complex, multi‐step process that ideally follows the C1 pathway to fully convert ethanol into CO_2_ through a 12‐electron transfer pathway. However, the practical operation of EOR is hindered by slow reaction kinetics and susceptibility to the less efficient C2 pathway, which results in partially oxidized products, such as acetaldehyde and acetic acid [5]. To overcome the kinetic and selectivity limitations of conventional catalysts, significant research efforts have been focused on designing electrocatalysts that not only accelerate C─C bond cleavage, but also steer the reaction preferentially toward the C1 route. Among noble metals, Pd and Pt are well‐established for their high EOR reactivity, especially in alkaline media, owing to their favorable adsorption characteristics and moderate oxophilicity. Nevertheless, the inherent C1 selectivity of state‐of‐the‐art Pd or Pt‐based nanocrystals remains unsatisfactory, typically below 7.5%, due to poisoning intermediates and inefficient C─C bond cleavage [6, 7, 8, 9]. Recent strategies to enhance catalyst selectivity and suppress poisoning species emphasize precise atomic‐level structural control. Facet‐dependent studies have been employed to tune intermediate binding energies and modulate reaction pathways [10, 11, 12]. Meanwhile, high‐entropy alloys (HEAs), multi‐element systems with diverse local environments, have emerged as a powerful design platform for complex reactions by alleviating scaling relations through multiple adsorption sites. Synergistic electronic interactions among constituent elements, together with sluggish diffusion, generate unique active sites, and improved structural stability, enabling enhanced activity and durability under the harsh electrochemical conditions of ethanol oxidation [13, 14, 15]. However, their complex composition and limited facet‐controlling synthetic strategies for preparing HEA catalysts pose a significant challenge in understanding the interaction between surface facets, elemental migration, and the dynamic evolution of structure‐activity‐stability relationships during operation.

Herein, we show that the facet‐controlled Pd@HEA core‐shell nanocrystals undergo electrochemically induced structural evolution during EOR, with the transformation of the structure from original cubic morphologies with a predominant {100} facet into severely beveled cubes with a balanced distribution of {100}, {110}, and {111} facets. This facet reorganization, where edge truncation generates more open {110}‐like motifs, while corner truncation and local surface‐energy minimization favor the emergence of more stable {111} facets, substantially enhances catalytic performance. The optimized structure achieves a forward peak specific activity of 12.4 A F^−1^, an 8.3‐fold enhancement compared to the initial activity, accompanied by a reduction in forward peak potential at 0.768 V (all potentials are referenced versus RHE unless otherwise stated). Importantly, the optimized HEA catalysts display a high forward‐to‐backward peak current density ratio (*j*_f_/*j*_b_) of 2.01, indicating the efficient oxidation of ethanol and the suppression of poisoning intermediates during the reaction process [14, 16]. These metrics highlight the outstanding performance of our Pd@HEA catalyst, positioning it among the most efficient platinum‐group‐metal (PGM)‐based systems reported to date. In situ surface‐enhanced infrared absorption spectroscopy (SEIRAS), electrochemical stripping tests, and computational calculations demonstrate that enhanced performance arises from a dual mechanism: (i) Accelerated oxidation of key reaction intermediates and (ii) effective suppression of the surface poisoning by strongly adsorbed bidentate HCOO_ad_. The optimized Pd@HEA catalysts exhibit a nearly complete selectivity toward the C1 pathway, in contrast to single‐metallic Pd nanocubes, which involve a mixed C1/C2 pathway. Moreover, the core‐shell nanocrystals retain a specific activity with negligible loss (at 0.77 V in cyclic voltammetry (CV) scans) even after extended chronoamperometric testing, while Pd nanocubes lose 53% activity. Overall, this work establishes a clear link between electrochemically induced structural evolution, elemental synergy, and sustained high C1 selectivity in EOR. These insights deepen the mechanistic understanding of HEA catalysts and guide next‐generation catalyst design.

## 2 Results and Discussion

### 2.1 Electrochemical EOR Activity of Pd@Pd_0.2_Pt_0.2_Ir_0.2_Ru_0.2_Rh_0.2_ Core‐Shell Nanocrystals

**FIGURE 1:**
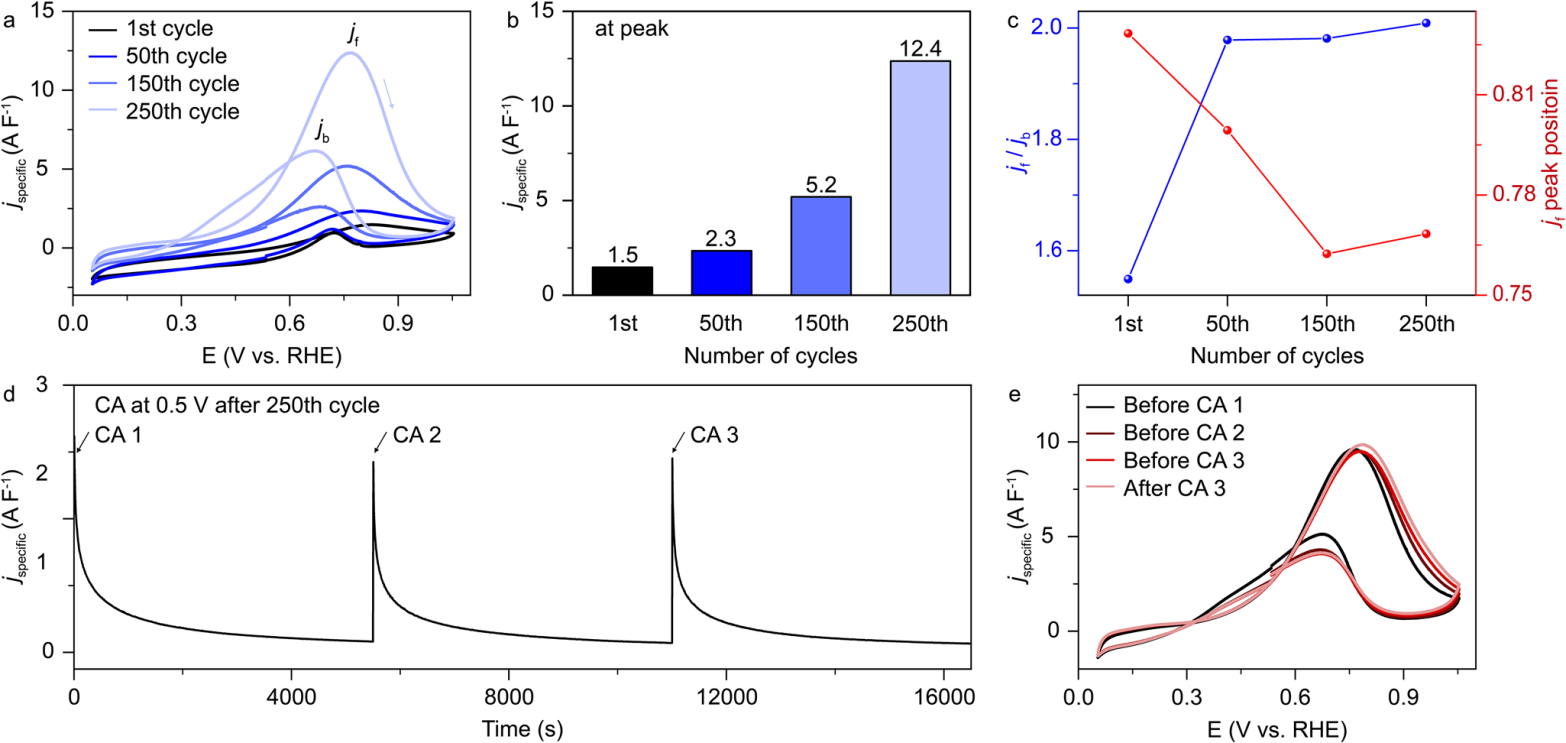
Electrochemical EOR performance of Pd@Pd_0.2_Pt_0.2_Ir_0.2_Ru_0.2_Rh_0.2_ core‐shell nanocrystals. Specific current densities are calculated by directly dividing the current measured by the surface capacitance in Farads. (a) ECSA‐normalized CV profiles, measured from 0.05 to 1.05 V versus RHE at a scan rate of 50 mV·s^−1^, obtained at the first, 50^th^, 150^th^, and 250^th^ cycles in Ar‐saturated 1 M KOH + 1 M ethanol. (b) The specific activities at the forward peak current (*j*_f_). (c) *j*_f_/*j*_b_ ratio and the potential position of the *j*_f_ peak. (d) CA tests at 0.5 V versus RHE after 250 EOR cycles. The arrow indicates the start of CA and CVs are collected before or after these indicated points. (e) The CV profiles obtained during the panel (d) experiment, either before or after CA tests.

The Pd@Pd_0.2_Pt_0.2_Ir_0.2_Ru_0.2_Rh_0.2_ core‐shell nanocrystals were prepared by layer‐by‐layer epitaxial growth, depositing 3–5 atomic layers of a Pd‐Pt‐Ir‐Ru‐Rh HEA onto Pd nanocubes (ca. 15 nm, Figure S1). The resulting solid‐solution HEA shell preserves the cubic morphology and exposed facets. The Pd core was deliberately chosen as a well‐defined cubic template because its close lattice matching with Pd, Pt, Ir, Ru, and Rh facilitates conformal epitaxial growth of the multi‐metallic shell (Pd/Pd = 0%; Pd/Pt = 0.51%; Pd/Ir = 1.79%; Pd/Ru = 3.32%; Pd/Rh = 2.81%) [17]. At the same time, Pd is intrinsically active for ethanol oxidation in alkaline media, while the PGM‐HEA shell introduces atomic‐level mixing and synergistic multifunctional sites that could further optimize intermediate oxidation, C─C bond cleavage, and catalyst stability. Detailed synthetic procedures are described in our previous work [18], and in the Experimental Section. To evaluate EOR activity over electroanalytical cycles, CV measurements were performed using Pd@Pd_0.2_Pt_0.2_Ir_0.2_Ru_0.2_Rh_0.2_ core‐shell nanocrystals in an Ar‐saturated 1 M KOH + 1 M ethanol electrolyte. Measurements employed a standard three‐electrode configuration at 50 mV s^−1^ over 0.05–1.05 V versus RHE. CVs collected at the first, 50^th^, 150^th^, and 250^th^ cycles were normalized by the electrochemically active surface area (ECSA, Supporting Information) to assess intrinsic activity (Figures 1a, S2, and S3). The estimation of an absolute ECSA of an HEA by CV is challenging due to the unknown specific capacitance of the HEA. Similarly, stripping (e.g., CO) measurements provide a relative measure, but as the charge associated with CO oxidation depends strongly on the local multielement surface environment, absolute measures are not achievable. Therefore, ECSA‐corrected current densities are presented by normalizing current to surface capacitance measured by CV, and trends were cross‐validated with CO stripping measurements (Figures S2 and S3).

All cycles exhibit two anodic peaks: a forward peak (*j*_f_) corresponding to ethanol oxidation, and a smaller backward peak (*j*_b_), attributed to the oxidation of residual carbonaceous intermediates [19]. Both *j*_f_ and *j*_b_ have progressively increased over cycles, with *j*_f_ reaching a maximum of 12.4 A F^−1^ at the 250^th^ cycle, which is 8.3, 5.4, and 2.4 times higher than those at the 1^st^, 50^th^, and 150^th^ cycles, respectively (Figure 1b). Two key performance metrics were analyzed (Figure 1c). The *j*_f_/*j*_b_ ratio increased from 1.55 (1^st^ cycle) to 2.01 (250^th^ cycle), suggesting enhanced forward oxidation and suppressed accumulation of poisoning species [14, 16]. Additionally, the forward peak potential shifted negatively from 0.838 V (1^st^) to 0.799 V (50^th^), 0.762 V (150^th^), and 0.768 V (250^th^), which is favorable for practical fuel cell operation by enlarging the potential gap between EOR and the counterpart oxygen reduction reaction (ORR) [9, 20]. In addition, onset potentials (defined at 5% of the *j*_f_) further confirm that the 250^th^‐cycle catalyst exhibits one of the lowest onset potentials among reported PGM‐based EOR catalysts (Table S1), reflecting facile ethanol activation and rapid intermediate conversion. Beyond 250 CV cycles, catalytic performance declines: While the *j*_f_ value of the 500^th^ cycle remains comparable with that of the 250^th^, the *j*_f_/*j*_b_ ratio of the 500^th^ cycle has dropped to 1.14, and the *j*_f_ potential has shifted to 0.874 V, confirming that optimal performance is achieved at around 250 cycles (Figure S4).

To assess operational stability, the Pd@Pd_0.2_Pt_0.2_Ir_0.2_Ru_0.2_Rh_0.2_ core‐shell nanocrystals optimized after the 250^th^ CV cycle was subjected to three consecutive chronoamperometry (CA) tests at 0.5 V for 5500 s each (Figure 1d). After brief intervening CV scans, the current recovered reproducibly, retaining ∼81% of its initial value at 5500 s (0.16 and 0.13 A F^−1^ at the end of the first and third CA (CA1 and CA3, respectively)). Furthermore, the CV profile acquired after CA testing closely matches the CV acquired before the first CA test (i.e., this CV is identical to the 250^th^ EOR CV, Figure 1e), indicating preserved intrinsic activity during CA testing, in contrast to prolonged CV cycling that drives the structural changes. In an individual CA, an initial drop in current upon polarization can be attributed in part to capacitive current decay, but more significantly, surface blockage by the intermediates formed as a result of incomplete oxidation at the constantly low applied potential (0.5 V); however, this was effectively recovered by two brief CV cycling. Consistent with this behavior, an extended CA test for 40, 000 s followed by an additional 5000 s CA measurement after a brief CV refresh again showed reproducible current recovery and nearly unchanged CV profiles before and after CA, further demonstrating the excellent long‐term electrochemical stability of the optimized catalyst (Figure S5a,b).

For comparison, Pd nanocubes without the HEA shell were evaluated (Figures S6 and S7). In the case of Pd nanocubes, unlike the HEA catalyst, an increase in EOR current was observed over a broader potential range with a less defined forward peak, which indicates the copresence of multiple reaction pathways and reduced selectivity. After 250 cycles, specific activity at 0.77 V decreased to 28% of the initial value (from 8.8 to 2.4 A F^−1^, Figure S6b), while the HEA catalyst increased by 954% (from 1.3 to 12.4 A F^−1^, Figure 1b). Three CA tests were also conducted using Pd nanocubes tested after a single CV cycle, where their EOR performance was optimal. Compared to CA1, the steady‐state current decreased to 72% in CA3 (Figure S6c), while the specific activity at 0.77 V in CV scans recorded after each CA test decreased to 59%, 53%, and 47%, respectively (Figure S6b,d). High‐angle annular dark‐field scanning transmission electron microscopy (HAADF‐STEM) and energy‐dispersive spectroscopy (EDS) elemental mapping after 250 EOR cycles further revealed surface oxidation and particle aggregation for Pd nanocubes, supporting catalyst deactivation through Pd oxidation (oxidation onset ∼0.95 V), surface aggregation, and intermediate poisoning (Figure S8).

To examine CO tolerance, CV measurements in CO‐saturated electrolyte were conducted (Figure S9) [8, 21]. For the Pd@Pd_0.2_Pt_0.2_Ir_0.2_Ru_0.2_Rh_0.2_ core‐shell nanocrystals, a broad and continuous desorption feature was observed from the first through 250^th^ cycles, reflecting diverse adsorption sites on the catalyst. The consistent CV shape, accompanied by a slight increase in current, suggests stable CO binding strength and a growing number of accessible active sites for CO oxidation. In contrast, Pd nanocubes initially exhibited a single, sharp CO stripping peak that progressively weakened and shifted to higher potentials with cycling, indicating loss of active sites and reduced CO oxidation activity. Overall, the entropy‐stabilized, multifunctional surface of the Pd@Pd_0.2_Pt_0.2_Ir_0.2_Ru_0.2_Rh_0.2_ core‐shell nanocrystals deliver superior durability, oxidation resistance, and anti‐poisoning capability, conferring clear advantages over monometallic Pd for long‐term electrocatalytic applications.

### 2.2 Structural Evolution of the Pd@Pd_0.2_Pt_0.2_Ir_0.2_Ru_0.2_Rh_0.2_ Core‐Shell Nanocrystals During the EOR

**FIGURE 2:**
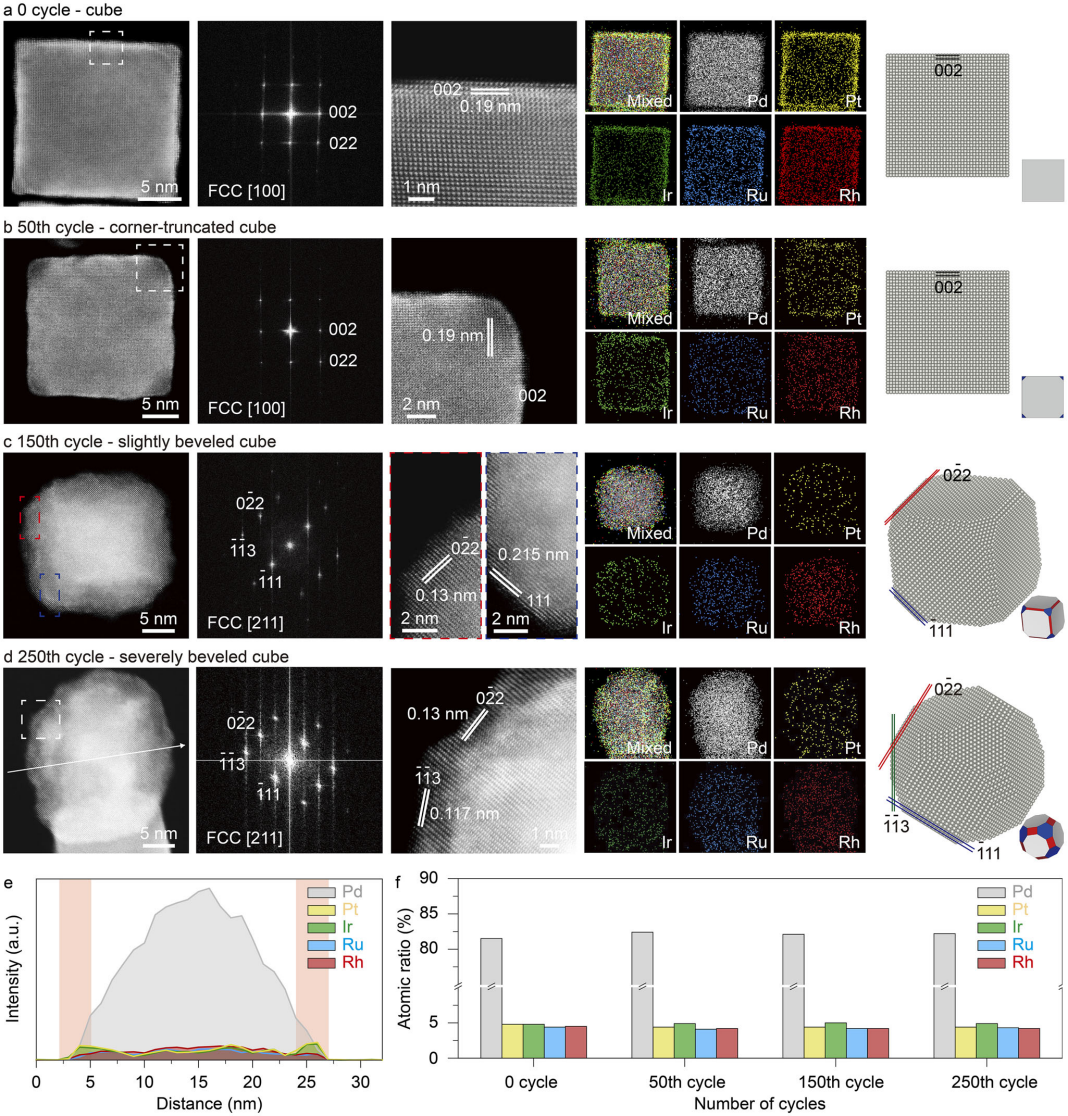
Characterization of Pd@Pd_0.2_Pt_0.2_Ir_0.2_Ru_0.2_Rh_0.2_ core‐shell nanocrystals after 0 (pristine sample), 50, 150, and 250 EOR cycles. (a‐d) HAADF‐STEM images, corresponding FFT patterns, EDS elemental maps, and schematic facet orientation illustrations for nanocrystals after (a) 0, (b) 50, (c) 150, and (d) 250 EOR cycles. (e) EDS line‐scan analysis for the sample after 250 cycles, taken along the arrow direction shown in (d). (f) Elemental composition determined using ICP‐OES.

To monitor the structural evolution of Pd@Pd_0.2_Pt_0.2_Ir_0.2_Ru_0.2_Rh_0.2_ core‐shell nanocrystals under EOR conditions, a series of advanced characterization techniques were conducted before electrochemical testing and after 50, 150, 250, and 500 EOR cycles, including atomic‐resolution HAADF‐STEM, fast Fourier transform (FFT) diffractograms, EDS elemental mapping, line scan analysis, schematic orientation illustrations, and inductively coupled plasma optical emission spectrometry (ICP‐OES) (Figures 2, S10, and Table S2).

The pristine Pd@Pd_0.2_Pt_0.2_Ir_0.2_Ru_0.2_Rh_0.2_ core‐shell nanocrystals exhibit well‐defined cubic morphology as shown by HAADF‐STEM imaging (Figure 2a). The contrast between the core and shell regions highlights a difference in atomic number. The FFT diffractogram along the [100] zone axis exhibits a single set of face‐centered cubic (FCC) diffraction spots, indicating phase purity and crystallinity. High‐resolution images display an atomically smooth surface with lattice fringes spaced at approximately 0.19 nm, corresponding to the (200) plane, thereby confirming predominant {100} facets, as highlighted in grey in the smaller schematic depiction (i.e., the right‐most image in the figure panel). In addition, EDS mapping shows uniform distributions of Pd, Pt, Ir, Ru, and Rh in the shell, with no detectable segregation. After 50 EOR cycles (Figure 2b), the nanocrystals exhibit slight truncation at the corners, evolving into mildly rounded cubes while retaining a single‐phase FCC structure, with predominant {100} surface coverage. After 150 cycles (Figure 2c), truncation becomes more pronounced at the corners and edges, leading to the formation of twelve {110} facets and eight {111} facets, which are marked in red and blue, respectively, in the schematic illustration. Corresponding d‐spacings of ∼0.13 nm and ∼0.22 nm are observed at the truncated edges and corners, respectively, consistent with {110} and {111} planes. After 250 cycles (Figure 2d,e), morphology evolves into a severely beveled cubic shape, with substantially increased exposure of {110} and {111} facets. EDS mapping and line scans confirm continued homogeneous distribution of all five constituent elements. ICP‐OES analysis shows negligible compositional changes from pristine to 250 cycles, indicating excellent chemical robustness (Figure 2f and Table S2).

**FIGURE 3:**
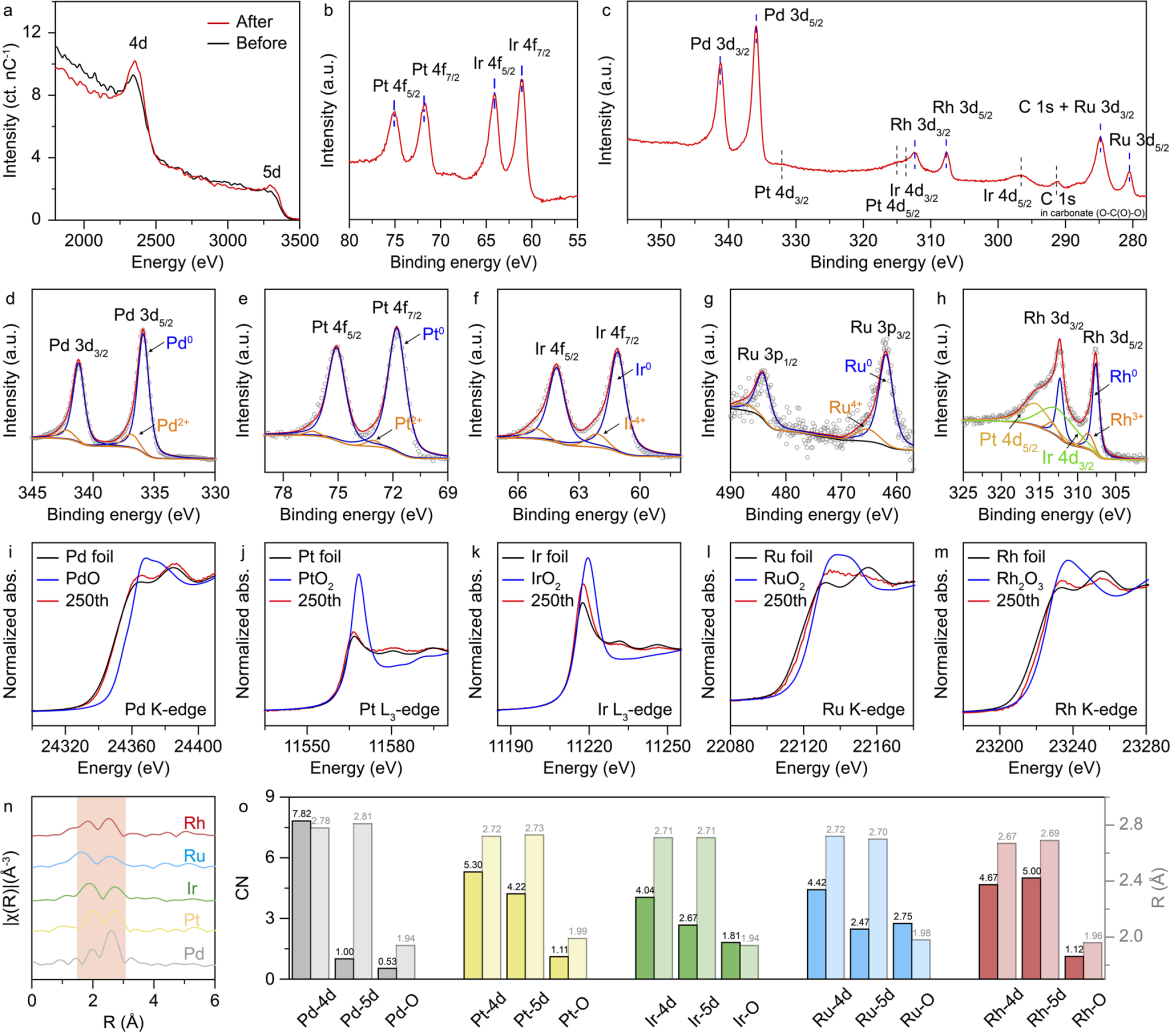
Surface chemical states and atomic‐scale mixing analysis of Pd@Pd_0.2_Pt_0.2_Ir_0.2_Ru_0.2_Rh_0.2_ core‐shell nanocrystals after 250 EOR cycles. (a) LEIS spectra are shown alongside the pre‐electrocatalysis sample (black line) for comparison. (b, c) High‐resolution XPS survey scans covering (b) 55–80 eV and (c) 278–355 eV. XPS fitted peaks are presented for (d) Pd 3d, (e) Pt 4f, (f) Ir 4f, (g) Ru 3p, and (h) Rh 3d. (i‐m) XANES profiles and corresponding reference standards for (i) Pd K‐edge, (j) Pt L_3_‐edge, (k) Ir L_3_‐edge, (l) Ru K‐edge, and (m) Rh K‐edge. (n) FT‐EXAFS spectra and (o) corresponding fitting results, providing coordination numbers (CN) and radial distances (R).

The surface chemical environments of the HEA shell in Pd@Pd_0.2_Pt_0.2_Ir_0.2_Ru_0.2_Rh_0.2_ core‐shell nanocrystals, before and after 250 EOR cycles, were investigated via low‐energy ion scattering (LEIS) analysis (Figures 3a and S11). The integrated peak area ratios of light 4d elements (Pd, Ru, Rh) to heavy 5d elements (Pt, Ir) remain nearly unchanged, with average values of 3.8 ± 0.8 (pre‐EOR) and 4.3 ± 0.8 (post‐EOR). This indicates that the elemental distribution in the outermost atomic layers of the HEA remains virtually unaltered. In the x‐ray photoelectron spectroscopy (XPS) spectra, characteristic 4f doublet peaks of Ir and Pt are clearly observed in the 55–80 eV region (Figure 3b), while Ru, Rh, and Pd 3d doublets are detected between 278 and 355 eV, along with weaker Pt 4d and Ir 4d signals (Figure 3c). Notably, these post‐cycling spectra retain similar peak positions and profiles to the pre‐EOR state (Figure S12) and the reference bulk values [19, 22]. These observations further indicate that all five elements remain present at or near the catalyst surface after extended cycling. Additionally, a distinct signal near 291 eV in the C 1s region emerges after cycling, ascribable to the formation of carbonate species (CO_3_^2−^) formed during CO_2_ evolution in EOR (Figure 3c). To deconvolute the chemical states, high‐resolution fitting was performed for each element (Figures 3d–h and S12c–g). As summarized in Table S3, the metallic‐to‐oxidized state ratios for Pd^0^/Pd^2+^, Pt^0^/Pt^2+^, Ir^0^/Ir^4+^, Ru^0^/Ru^4+^, and Rh^0^/Rh^3+^ after 250 cycles were determined to be 87.9/12.1, 96.8/3.2, 88.3/11.7, 84.8/15.2, and 79.3/20.7, respectively. These values are comparable to pre‐cycling values (91.2/8.8, 95.9/4.1, 89.4/10.6, 88.0/12.0, and 80.9/19.1), suggesting that the HEA shell retains its predominantly metallic character with minimal surface oxidation. Owing to the overlap between Ru 3d and C 1s signals, Ru 3p peaks were selected for Ru deconvolution.

Synchrotron‐based x‐ray absorption spectroscopy (XAS) was utilized to investigate the electronic structure and local coordination environments of the Pd@Pd_0.2_Pt_0.2_Ir_0.2_Ru_0.2_Rh_0.2_ core‐shell nanocrystals after 250 EOR cycles. X‐ray absorption near‐edge structure (XANES) spectra for all constituent elements are presented in Figure 3i–m, together with their corresponding metal and oxide references. The absorption edges of the post‐EOR sample align closely with those of metal foils; however, distinct oscillation features are observed relative to pure metals, indicating that the 4d and 5d metals within the HEA shell largely maintain their metallic character, but possess modified local atomic and electronic structures. Fourier‐transform extended x‐ray absorption fine structure (FT‐EXAFS) spectra (Figures 3n and S13) display distinct alloy‐related doublet peaks in all of the studied elements, indicative of strong atomic interactions between the constituent elements [23]. Furthermore, the radial bond distances of the 5d elements (Pt and Ir) are slightly longer than those of the 4d elements (Pd, Ru, and Rh), in agreement with their larger atomic radii, which evidences the presence of an HEA phase. Detailed fitting data and parameters for FT‐EXAFS are provided in Figures 3o, S14, S15, and Table S4. The derived coordination numbers (CN) for neighboring 4d (Pd, Ru, Rh) and 5d (Pt, Ir) atoms surrounding the target elements can reveal the presence of extensive atomic mixing (Figure 3o and Table S4). Specifically, the M‐4d/M‐5d coordination values for Pt, Ir, Ru, and Rh were determined to be 5.30/4.22, 4.04/2.67, 4.42/2.47, and 4.67/5.00, respectively, demonstrating substantial intermixing between the 4d and 5d elements with no discernible elemental segregation. In contrast, Pd exhibits a notably higher Pd‐Pd coordination (M‐4d/M‐5d = 7.82/1.00), consistent with a Pd‐rich core beneath the multi‐metallic HEA shell. Furthermore, in all elements, the CNs corresponding to metallic bonds are considerably higher than those associated with metal‐oxygen coordination, with oxidized species (M─O) accounting for less than 30% of total CNs. In addition, x‐ray diffraction (XRD) patterns (Figure S16) reveal that the Pd@Pd_0.2_Pt_0.2_Ir_0.2_Ru_0.2_Rh_0.2_ core‐shell nanocrystals maintain a consistent FCC structure before and after EOR cycling. Consistent with these post‐cycling results, post‐CA characterization of the catalyst, after 250 EOR cycles followed by prolonged CA operation for 45, 000 s at 0.5 V, further confirms its excellent structural robustness (Figure S5, Tables S2, S3, and S5). XRD patterns still show the characteristic FCC reflections without detectable impurity phases or phase segregation, while HAADF‐STEM and FFT analyses verify that the severely beveled cubic morphology with co‐exposed {100}, {110}, and {111} facets is well preserved. EDS mapping and ICP‐OES further indicate a homogeneous elemental distribution and negligible compositional change after CA testing. Moreover, the post‐CA XPS spectra and fitted oxidation‐state ratios remain highly similar to those of the pre‐EOR and post‐cycling samples, indicating that the HEA shell largely retains its metallic character with only minor surface oxidation. These results demonstrate that the HEA shell remains structurally and chemically stable under prolonged potentiostatic EOR operation, while the morphology evolution is associated with repeated CV cycling rather than with CA testing.

In summary, during EOR cycling in alkaline media, the initially cubic Pd@Pd_0.2_Pt_0.2_Ir_0.2_Ru_0.2_Rh_0.2_ core‐shell nanocrystals evolve from a {100}‐dominated morphology into severely beveled cubes exposing a more balanced distribution of {100}, {110}, and {111} facets. This evolution can be understood as a potential‐driven surface reconstruction process initiated at the high‐energy corners and edges of the original cubes. The transformation is likely promoted by preferential OH^−^ adsorption at low‐coordination step atoms, where OH_ad_ can form at lower potentials than on terrace sites, rendering corner and edge atoms particularly susceptible to electrochemical activation. Repeated oxidation/reduction cycling can therefore destabilize these low‐coordination atoms, promote atom migration, and progressively truncate the sharp cubic geometry. In this process, edge truncation introduces more open {110}‐like surface motifs, whereas corner truncation and local surface‐energy minimization favor the emergence of more stable {111} facets, ultimately yielding a mixed‐facet, severely beveled morphology rather than a single‐facet reconstruction. This interpretation is consistent with previous reports on Pt‐family nanocubes and other shape‐controlled metallic catalysts, in which electrochemical cycling induces rounding/beveling and surface roughening [24, 25, 26, 27]. Importantly, our CO stripping results show a progressive negative shift of the stripping peak from the first to the 250^th^ cycle, indicating that OH_ad_ becomes available at lower potentials on the evolved surface (Figure S3a). Thus, the transition from cubes to severely beveled cubes is accompanied by increased accessibility of OH‐related reactive sites, which can facilitate the oxidation and removal of absorbed CO‐like intermediates during EOR [28, 29].

### 2.3 Investigation of Reaction Pathways and Intermediates During EOR

To elucidate EOR mechanisms on Pd@Pd_0.2_Pt_0.2_Ir_0.2_Ru_0.2_Rh_0.2_ core‐shell nanocrystals, in situ surface‐enhanced infrared absorption spectroscopy (SEIRAS) was employed [30]. Spectra were recorded with the application of constant potentials in a stepwise manner, with increments or decrements of 0.05 V over the potential range of 0.1 V to 1.05 V and back to 0.1 V versus RHE. A change in infrared absorbance relative to the signals obtained at the first forward step (i.e., 0.1 V vs. RHE) is plotted in the reported spectra. Positive and negative absorption bands correspond to the formation and depletion of molecular species, respectively. To analyze the dynamic spectral changes, perturbation‐correlation moving window two‐dimensional (PCMW2D) correlation spectroscopy was applied, enabling the deconvolution of overlapped peaks and distinguishing different phases of spectral variation [31, 32]. Band assignments were validated using control electrolytes containing representative EOR products and intermediates (e.g., CH_3_COOH, HCOOH, CH_3_CHO, and C_2_H_5_OH) in 1 M KOH (Figure S17 and Table S6) [33, 34, 35, 36].

**FIGURE 4:**
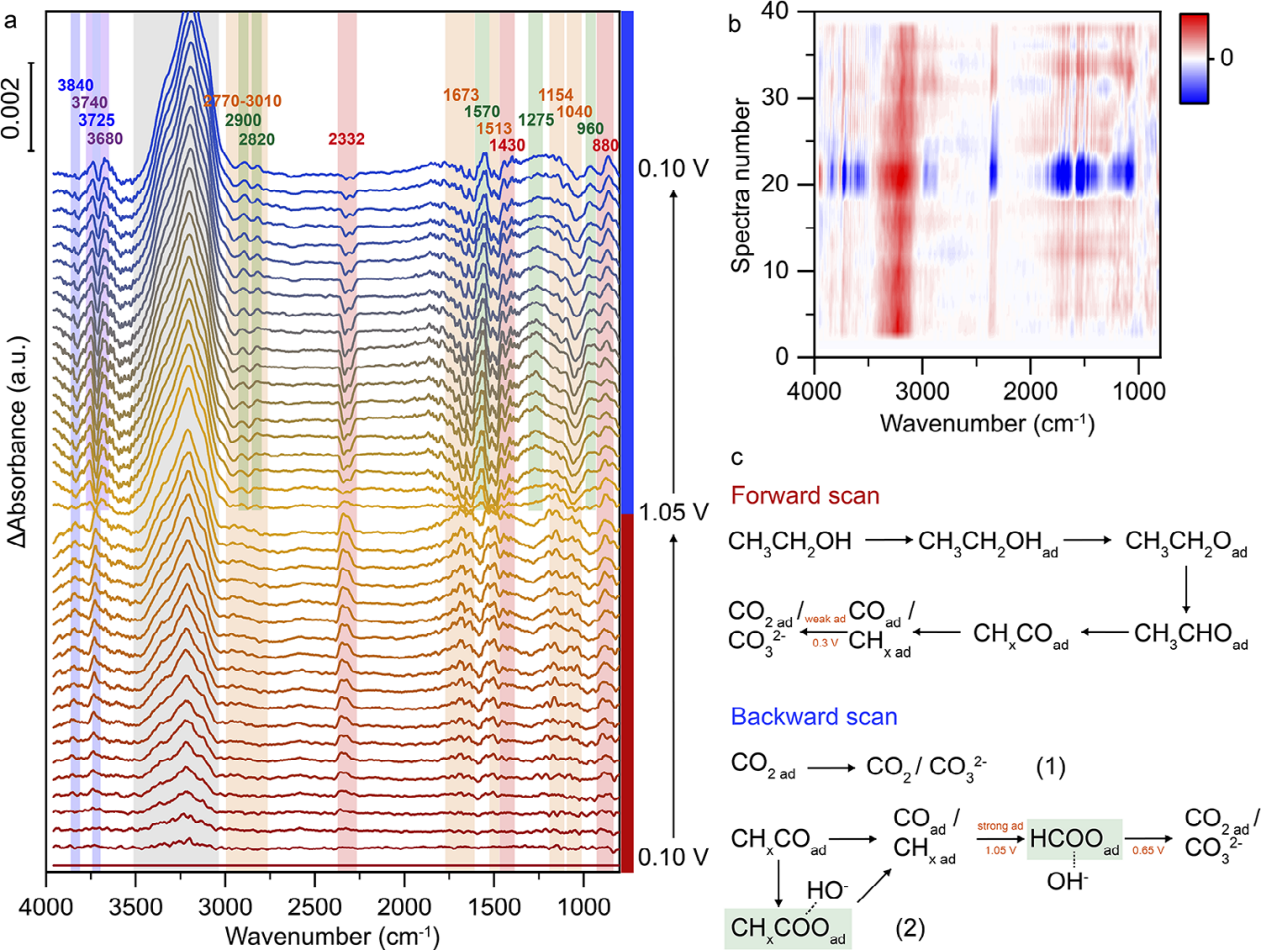
In situ SEIRAS spectral analysis during the first EOR cycle for Pd@Pd_0.2_Pt_0.2_Ir_0.2_Ru_0.2_Rh_0.2_ core‐shell nanocrystals in 1 M KOH + 1 M ethanol (Ar‐saturated) electrolyte. (a) SEIRAS spectra collected with a potential step of 0.05 V at a potential window of 0.1‐1.05 V versus RHE. (b) Synchronous PCMW2D correlation map highlighting dynamic changes in absorption during potential sweeps. (c) Proposed EOR pathway on Pd@Pd_0.2_Pt_0.2_Ir_0.2_Ru_0.2_Rh_0.2_ core‐shell nanocrystals during the first cycle.

Figures 4 and S18a were recorded after completion of the first EOR cycle, where the catalyst morphology is a cube. With increasing potential, adsorption, and oxidation of ethanol, ethoxy, and acetyl species intensify, as indicated by the orange region (∼1040, 1154, 1513, 1673, and 2770–3010 cm^−1^, see Table S6). A signature υ(CO_2_) band for adsorbed CO_2_ at 2332 cm^−1^ appears at low potentials (>0.3 V), indicating the early onset of C1 oxidation products and efficient C─C bond cleavage. The subsequent reaction with OH^−^ yields carbonate species (CO_3_^2−^), with characteristic bands at 1430 and 880 cm^−1^ (red shading). Simultaneously, intense OH^−^ adsorption bands at 3725 and 3840 cm^−1^ reflect the oxophilic nature of the surface. Notably, no CO adsorption bands were detected, indicating rapid oxidation to CO_2_ without surface poisoning. Upon reversing the scan direction, bands corresponding to ethanol, ethoxy, acetyl (∼1040, 1154, 1513, 1673, and 2770–3010 cm^−1^, orange shading), CO_2_ (∼2332 cm^−1^), and OH^−^ (∼3725 and 3840 cm^−1^, blue shading) become negative, while a new group of competitor species, mainly ascribable to carboxylate species (e.g., HCOO_ad_ or minor CH_3_COO_ad_), emerges with the signature asymmetric stretching vibrations of COO at 1570 cm^−1^ (green shading). The appearance of negative bands in Figure 4a arises from pre‐adsorbed intermediates retained following a preliminary CV scan, which ensured comparable surface conditions for subsequent SEIRAS measurements. Further experimental details are provided in the Experimental Section. The observation of a new group of surface intermediates at the start of the reverse scan (1.05 to 0.65 V) shows that the catalyst was unable to oxidize strongly adsorbed residual intermediates which had arisen from the preceding forward scan, resulting in the accumulation of competitor species rather than complete conversion to CO_2_. The continued presence of CO_3_^2−^ bands indicate partial desorption of remaining CO_2 ad_ into the alkaline electrolyte. As strongly adsorbed carboxylates replace OH^−^ on the surface, negative bands at 3725 and 3840 cm^−1^ emerge, while new bands arise at 3680 and 3740 cm^−1^ (purple shading in Figure 4a, also seen in the synchronous PCMW2D correlation map (spectra 18 to 24, Figure 4b)) can be assigned to weakly hydrogen‐bonded OH^−^ interacting with adsorbed carboxylates. During the reverse scan, at ∼0.65 V, where it coincides with the onset of the backward current peak (*j*_b_), the CO_2_ band begins to recover (becomes less negative in the difference spectra), together with the reinitiation of ethanol adsorption (orange shading) and a decline in competitor bands (green shading). This suggests the regeneration of active sites for the EOR through either the oxidation of the strongly absorbed intermediates or their potential‐driven desorption from the electrode surface at less positive applied potentials.

After 50 CV cycles (Figures S19 and S18b), when the catalyst morphology evolves into corner‐truncated cubes, the forward scan remains dominated by the C1 pathway and CO_2_ formation up to ∼0.95 V. However, blocking intermediates, such as HCOO_ad_ or minor CH_3_COO_ad_ begins to appear (see green shaded region, Figure S19) at earlier potentials compared to the first cycle, which is now around 0.95 V. During the reverse scan, CO_2_ regeneration and ethanol re‐adsorption also occur at earlier potentials (∼0.75 V), and fewer competitor species are observed at the end of the cycle, suggesting a “cleaner” surface. This trend becomes more pronounced at the 150^th^ cycle (slightly beveled cubes, Figures S20 and S18c). Competitor species formation now begins at around 0.75 V, closely matching the forward peak potential (*j*_f_), thus narrowing the potential window in the forward scan for CO_2_ generation. Nonetheless, the regeneration of active sites and the production of CO_2_ resume at earlier potentials in the reverse scan, closer to 1.05 V. Although increasing the number of cycles from 1 to 150 has increased surface reactivity, it also accelerates the formation of “blocking” carboxyl species. However, the catalyst also becomes increasingly effective upon the removal of blocking species in the backward scan. These observations are consistent with the EOR electrochemical activity measured by CV. In that it exhibits a shift toward an earlier occurrence of *j*_f_ potentials and a broadened current window in the backward scan.

**FIGURE 5:**
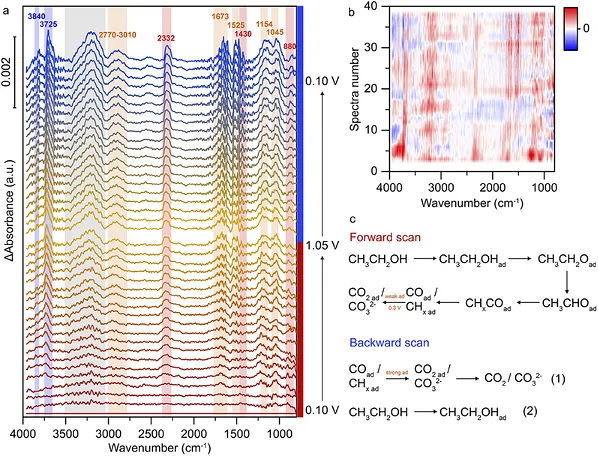
In situ SEIRAS spectral analysis during the 250th EOR cycle for Pd@Pd_0.2_Pt_0.2_Ir_0.2_Ru_0.2_Rh_0.2_ core‐shell nanocrystals in 1 M KOH + 1 M ethanol (Ar‐saturated) electrolyte. (a) SEIRAS spectra collected with a potential step of 0.05 V at a potential window of 0.1‐1.05 V versus RHE. (b) Synchronous PCMW2D correlation map highlighting dynamic changes in absorption during potential sweeps. (c) Proposed EOR pathway on Pd@Pd_0.2_Pt_0.2_Ir_0.2_Ru_0.2_Rh_0.2_ core‐shell nanocrystals during the 250^th^ cycle.

At the 250^th^ cycle (Figures 5 and S18d), where the catalyst morphology is a severely beveled cube, the C1 oxidation pathway dominates throughout the entire potential range. The highly catalytic HEA surfaces have enabled complete ethanol oxidation at low potentials, with negligible accumulation of blocking species. This is demonstrated by the continued accumulation of ethanol, ethoxy, acetyl (∼1040, 1154, 1513, 1673, and 2770–3010 cm^−1^, orange shading), CO_2_ (∼2332 cm^−1^), and OH^−^ (∼3725 and 3840 cm^−1^, blue shading) at the catalyst surface (the bands do not go negative at any potential, in contrast to SEIRAS data of a catalyst which has not undergone the extensive CV cycling, Figure 4). The OH^−^ bands accumulate at lower (less positive) potentials on the sample that has undergone 250 CV cycles (blue shading, Figure 5), compared to during the first CV (Figure 4) in line with expectations from the CO stripping experiments (Figure S3a). A slight decrease in CO_2_ intensity is observed at high forward potentials (0.8–1.05 V), likely due to the overaccumulation of oxygenated species, which can hinder additional ethanol adsorption and oxidation, as evidenced by the intense OH^−^ adsorption bands at 3725 and 3840 cm^−1^. These species are reduced or reorganized during the reverse scan, leading to the reappearance of CO_2_ bands and restoration of catalytic activity [37].

Overall, in situ SEIRAS analysis reveals that the Pd@Pd_0.2_Pt_0.2_Ir_0.2_Ru_0.2_Rh_0.2_ core‐shell nanocrystals predominantly follow the C1 complete oxidation pathway, producing CO_2_ as the major and sole product while suppressing carboxylate accumulation as cycling proceeds. A similar series of SEIRAS studies is carried out on the Pd reference sample (see Supporting Information). In contrast, the SEIRAS of the reference Pd nanocubes shows the presence of products from both the C1/C2 oxidation pathway, generating acetaldehyde, acetate, and CO_2_ at different stages of the applied potential. The presence of CO‐related absorption bands (1900–2050 cm^−1^) further indicates the sluggishness of a complete oxidation process and the formation of strongly adsorbed CO species; it should be noted that those CO species are known to block active sites and hinder EOR performance (Figure S21) [8, 38, 39]. With continued cycling, the HEA catalyst increasingly accelerates the removal of poisoning intermediates, ultimately suppressing their formation after 250 cycles due to favorable morphological evolution. These spectroscopic results agree well with electrochemical data (Figure 1), demonstrating enhanced catalytic efficiency and reduced surface poisoning, as reflected by the increased *j*_f_/*j*_b_ ratio and negatively shifted *j*_f_ potential.

Electrochemical stripping experiments were conducted to provide in‐depth reaction profiles associated with various intermediates on the surfaces of Pd@Pd_0.2_Pt_0.2_Ir_0.2_Ru_0.2_Rh_0.2_ core‐shell nanocrystals at different morphological stages: Nanocubes (1^st^ cycle) and severely beveled cubes (250^th^ cycle). In each experiment, the catalysts were exposed to 1 M KOH electrolytes with 1 M of a probe intermediate (e.g., CH_3_CHO, HCOOH, and CH_3_COOH), followed by an initial stripping test, 250 EOR cycles in ethanol, and subsequent re‐testing after thorough rinsing. All the experiments were conducted individually using freshly prepared electrodes to avoid cross‐contamination. As shown in Figure S22, the oxidative current in 1 M KOH + 1 M CH_3_CHO is significantly enhanced in the 250^th^ cycle (severely beveled cubes) sample compared to the first cycle (cubes) sample. At 0.77 V (near the EOR forward peak, *j*_f_), the current of the severely beveled sample is 49.6% higher (Figure S22b), indicating an improved capacity for oxidizing acetyl‐like species—a key rate‐determining step in the C1 pathway involving C─C bond cleavage. The formic acid oxidation (FAO) profiles (Figure S23a) show that peaks below 0.55 V correspond to the direct oxidation of formate‐related intermediates into CO_2_ [40]. The severely beveled cube samples exhibit a 347.7% increase in peak current, indicating a markedly improved ability to convert HCOO_ad_ to CO_2_. This ability is particularly important as it has been demonstrated using SEIRAS that HCOO_ad_ acts as a key poisoning intermediate during the EOR process in our system. In contrast, electrochemical oxidation of CH_3_COOH is insignificant in both cubic and severely beveled catalysts (Figure S23b). Only weak adsorption of acetate was observed below 0.4 V (partially overlapping with hydrogen adsorption), followed by low‐intensity oxidation signals at potentials more positive than 0.8 V. These results imply that carboxylate poisoning observed between the first and 150^th^ EOR cycles is predominantly due to formate species (HCOO_ad_), rather than acetate (CH_3_COO_ad_), in agreement with SEIRAS band assignments.

**FIGURE 6:**
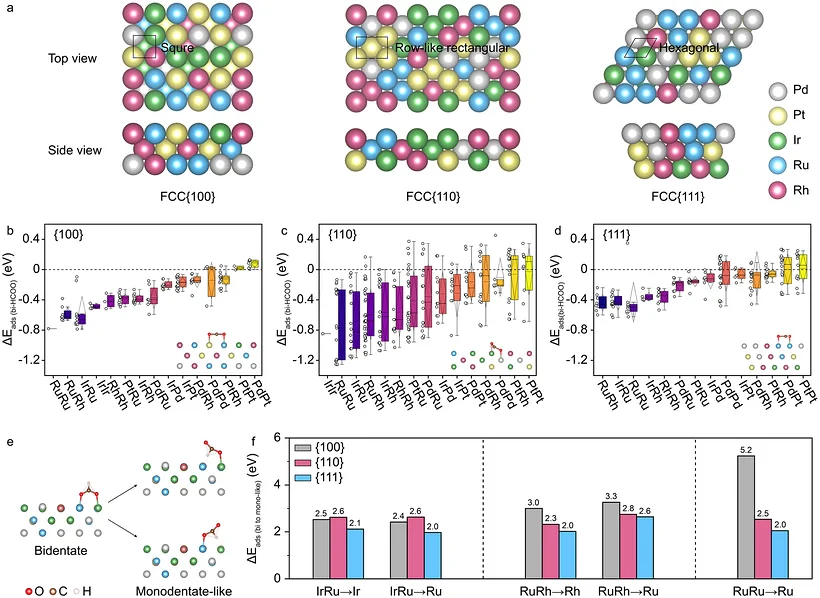
Computational investigation of HCOO adsorption on Pd_0.2_Pt_0.2_Ir_0.2_Ru_0.2_Rh_0.2_ HEA surfaces. (a) Top and side views of three‐layer FCC {100}, {110}, and {111} slab models. (b–d) Statistical distribution of bidentate HCOO_ad_ adsorption energies for different top‐top adsorption ensemble configurations on the {100}, {110}, and {111} facets, respectively, derived from MLIP calculations. (e) Schematic showing the conversion of bidentate HCOO_ad_ into a monodentate‐like configuration via cleavage of one O‐metal bond at the catalyst surface. (f) DFT‐calculated enthalpic costs associated with the bidentate‐to‐monodentate‐like transformation for the 15 strongest bidentate‐binding sites on each facet, including IrRu, RuRh, and RuRu ensembles.

To clarify the origin of suppressed HCOO_ad_ poisoning on the optimized HEA surface, we combined high‐throughput machine‐learning interatomic potential (MLIP) screening with density functional theory (DFT) analysis. Three highly mixed quasi‐random structure (SQS) slabs were selected for each facet, and all accessible top‐top bidentate HCOO_ad_ adsorption sites were evaluated (Figures 6a and S24). Here, we focus on the stability of adsorbates on each facet and future calculations could be used to validate the relative stability of the facets themselves. As shown in Figures 6b‐d, HEA{100} generally exhibited stronger bi‐HCOO_ad_ binding than HEA{111}, whereas HEA{110} showed a broader energy distribution due to greater structural flexibility. The strongest binding motifs were consistently Ru‐containing local ensembles, particularly RuRu, RuRh, and IrRu, identifying them as the most probable blocking sites for deeply bound bidentate formate. DFT calculations (Figures 6e,f, and S25) further revealed that the energy required to cleave one O‐metal bond and generate a mono‐like HCOO_ad_ configuration is significantly lower on the newly formed facets, especially HEA{111}. Because conversion of bi‐HCOO_ad_ to a mono‐like configuration is required for oxidative removal of strongly bound formate, these results show that facet evolution in the HEA mitigates persistent poisoning by promoting formate release and oxidation [41, 42, 43]. To further correlate these adsorption trends with the local electronic structure, facet‐dependent projected density of states (PDOS) analysis was performed. As seen in Figure S26, Ru‐, Rh‐, and Ir‐containing environments exhibit d states closer to the Fermi level, indicative stronger interactions with oxygen‐containing intermediates. Moreover, these metals on the {100} facet display an upshifted *d*‐band center and a more adsorption‐prone electronic structure than on the {110} and {111} facets [44, 45]. Consistently, HCOOH stripping tests on single‐metal and binary‐alloy core‐shell nanocrystals with strongly binding Ru‐based motifs (Pd@Ru, Pd@Ru_0.5_Rh_0.5_, and Pd@Ir_0.5_Ru_0.5_) show lower activity and higher stripping potentials than the Pd@Pd_0.2_Pt_0.2_Ir_0.2_Ru_0.2_Rh_0.2_ catalyst (Figure S27). Thus, suppression of HCOO_ad_ poisoning on the optimized HEA catalyst arises not from a single active element, but from a composition‐geometry synergy, in which the local elemental ensemble effect and facet evolution jointly regulates the stability and reactivity of formate‐related intermediates. Importantly, the retained single‐phase HEA shell stabilizes the multielement surface‐active sites during electrochemical reconstruction, thereby sustaining the enhanced ethanol oxidation activity.

## 3 Conclusion

We demonstrated that electrochemically induced facet optimization in Pd@Pd_0.2_Pt_0.2_Ir_0.2_Ru_0.2_Rh_0.2_ core‐shell nanocrystals generated a highly active and durable electrocatalyst for ethanol oxidation in alkaline media. The observed morphological evolution drives the transformation from a predominantly {100}‐terminated structure to a more stable configuration with balanced exposure of {100}, {110}, and {111} facets. Throughout this reconstruction, the nanocrystals maintain a single‐phase HEA solid solution structure. Electrocatalytic performance improves with continued CV cycling, reaching an optimal activity at around the 250^th^ cycle. At this stage, the nanocrystals exhibit a severely beveled cubic morphology with a maximized forward peak specific activity of 12.4 A F^−1^, an increased *j*_f_/*j*_b_ ratio of 2.01, and a reduced forward peak potential of 0.768 V, reflecting efficient ethanol oxidation and effective removal of poisoning intermediates. In situ SEIRAS reveals that the Pd@Pd_0.2_Pt_0.2_Ir_0.2_Ru_0.2_Rh_0.2_ core‐shell nanocrystals predominantly follow a complete C1 oxidation pathway, producing CO_2_ as the main product. This behavior is associated with the catalyst's increased ability to cleave the critical C─C bond and weaken poisoning intermediates, particularly bidentate HCOO_ad_, thereby mitigating surface deactivation and promoting more favorable reaction kinetics. By directly linking surface structure, intermediate dynamics, and catalytic performance, these findings advance the rational design of high‐entropy alloy catalysts for complex multi‐electron electrochemical reactions.

Supporting information for this article is given via a link at the end of the document (Methods, Figures S1 to S27, Tables S1 to S6).

## Author Contributions

A. J. C. and T. H. Y. conceived and supervised the research. Y. C. H. performed most of the experiments and data analysis. Y. C. H., H. J., A. R. N., L. J. H., and A. J. C. designed the SEIRAS experiment. R. J. P. conducted the ALD experiment. J. W. C. and Y. C. H. carried out the synchrotron XAS experiment and EXAFS fitting. Y. C. H. and J. T. L. performed detailed microscopic characterizations. A. M. G. conducted the LEIS experiments. Y. C. H. carried out, and K. H. L. supervised the computational calculations. Y. C. H. and K. F. L. conducted the synthesis of the Pd nanocubes. All authors discussed the results and contributed to the final manuscript.

## Conflicts of Interest

The authors declare no conflicts of interest.

## Supporting information



The authors have cited additional references within the Supporting Information [46, 47, 48, 49, 50, 51, 52, 53, 54, 55, 56, 57, 58, 59, 60, 61, 62, 63, 64, 65]. **Supporting File**: anie73583‐sup‐0001‐SuppMat.pdf.

## Data Availability

The data that support the findings of this study are available from the corresponding author upon reasonable request.
