# Supplementary material for: Electrochemically Induced Structural Evolution to Generate Optimized High‐Entropy‐Alloy Electrocatalysts for Ethanol Oxidation
Source: Angew Chem Int Ed Engl. 2026 Jul 11;65(38):e8871821. doi: 10.1002/anie.8871821 (PMC13573037; doi:10.1002/anie.8871821)
Supplement: Supplementary file 1 [file ANIE-65-e8871821-s001.pdf]

Supporting Information

Electrochemically Induced Structural Evolution to Generate Optimized High-Entropy-Alloy Electrocatalysts for Ethanol Oxidation

Yueh-Chun Hsiao,[1,2] Hansaem Jang,[1] Chun-Wei Chang,[2] Jui-Tai Lin,[2] Kuan-Fang Lee,[2] Adrian M. Gardner,[1,3,4] Richard J. Potter,[5] Alex R. Neale,[1] Laurence J. Hardwick,[1] Kun-Han Lin,[2] Tung-Han Yang[2,6,7,] Alexander J. Cowan,[1,]**

[1] Stephenson Institute for Renewable Energy and Department of Chemistry, University of Liverpool, Liverpool L69 7ZF, United Kingdom.

[2] Department of Chemical Engineering, National Tsing Hua University, Hsinchu 30013, Taiwan.

[3] Low Energy Ion Scattering Facility, George Holt Building, University of Liverpool, Brownlow Street, Liverpool, L69 3GB, United Kingdom.

[4] Early Career Laser Laboratory and Surface Science Research Centre, University of Liverpool, Liverpool L69 3BX, UK

[5] Department of Materials, Design and Manufacturing Engineering, University of Liverpool, Liverpool, L69 3GH, United Kingdom.

[6] High Entropy Materials Center, National Tsing Hua University, Hsinchu 30013, Taiwan.

[7]College of Semiconductor Research, National Tsing Hua University, Hsinchu 300044, Taiwan.

*Corresponding author. Email: acowan@liverpool.ac.uk; tunghanyang@mx.nthu.edu.tw

The PDF file includes: Methods, Figures S1 to S27, and Tables S1 to S6.

Methods

Chemicals: Sodium tetrachloropalladate (Na_2PdCl_4), potassium tetrachloroplatinate (K_2PtCl_4), dihydrogen hexachloroiridate ($H_2IrCl_6 \cdot xH_2O$), ruthenium chloride hydrate ($RuCl_3 \cdot xH_2O$), rhodium chloride hydrate ($RhCl_3 \cdot xH_2O$), poly(vinyl pyrrolidone) (PVP, $M_W \approx$ 55,000), *L*-ascorbic acid (AA), potassium hydroxide 99.99% (KOH), potassium bromide (KBr), 2-propanol ($(CH_3)_2CHOH$),formic acid 96% (HCOOH), and acetic acid 99.7% (CH_3COOH) were all obtained from Sigma-Aldrich. Ethylene glycol (EG), acetone 99.8% (CH_3COCH_3), and absolute ethanol 99.5% (C_2H_5OH) were purchased from J. T. Baker. Acetaldehyde 99.5% (CH_3CHO) was purchased from Thermo Scientific. Sigracet 39 BB gas diffusion layer (GDL) was purchased from SGL Carbon. Deionized (DI) water with a resistivity of 18.2 MΩ cm at 25 °C using Direct-Q® 3 UV water purification system, Merck, was used for all the experiments.

Synthesis of Pd Nanocube: Pd nanocubes with average edge lengths of 15.2 nm were prepared according to a previously reported method with slight modifications [46, 47]. In a 20-mL vial, an aqueous solution (8 mL) containing ascorbic acid (AA, 60 mg), poly(vinylpyrrolidone) (PVP, 105 mg), and potassium bromide (KBr, 600 mg) was heated to 80 °C under magnetic stirring for 10 min. Subsequently, 3 mL of an aqueous Na_2PdC_4 solution (57 mg) was rapidly added via pipette. The vial was then capped, and the reaction was maintained at 80 °C with magnetic stirring for 3 h. The resulting Pd nanocubes were collected by centrifugation, washed three times with DI water, and finally dispersed in EG for subsequent use.

Synthesis of Pd@PdPtIrRuRh Core-Shell Nanocrystals Using Epitaxial Growth: $Pd@Pd_{0.2}Pt_{0.2}Ir_{0.2}Ru_{0.2}Rh_{0.2}$ core-shell nanocrystals were synthesized via an epitaxial growth method. In a 20-mL vial, 0.40 mL of a suspension of Pd cubic seeds in EG, 20 mg of AA, 100 mg of PVP, 60 mg of KBr, and 3.1 mL of EG were combined. The mixture was heated at 110 °C for 15 min under magnetic stirring, and the temperature was subsequently raised to 195 °C over 30 min. A 14 mL precursor solution, containing equimolar amounts of Na_2PdCl_4, K_2PtCl_4, $H_2IrCl_6 \cdot xH_2O$, $RuCl_3 \cdot xH_2O$, and $RhCl_3 \cdot xH_2O$ in EG, was then titrated into the reaction solution at a rate of 0.8 mL h^{-1} using a syringe pump. The concentration of each metal precursor was set to 0.042 µmol mL^{-1} to achieve 3-5 atomic layers of the $Pd_{0.2}Pt_{0.2}Ir_{0.2}Ru_{0.2}Rh_{0.2}$ shell on the Pd seeds. Throughout the precursor addition, the reaction temperature was maintained at 195 °C. After the complete addition of the precursor solution, the reaction mixture was held at 195 °C for an additional 1 h. The as-synthesized $Pd@Pd_{0.2}Pt_{0.2}Ir_{0.2}Ru_{0.2}Rh_{0.2}$ core-shell nanocrystals were isolated by centrifugation, washed once with acetone, twice with DI water, and finally

redispersed in water for subsequent applications. For the Pd@Ru, Pd@$Ru_{0.5}Rh_{0.5}$, and Pd@$Ir_{0.5}Ru_{0.5}$ core-shell nanocrystals reference groups, a similar synthetic protocol was applied, while controlling the shell thickness within 3-5 atomic layers.

***Electrocatalytic Measurements for EOR*:** The electrochemical measurements were performed using a three-electrode cell connected to an Ivium Vertex electrochemical workstation. A glassy carbon electrode (ALS, 5 mm diameter) served as the working electrode, a Pt wire (ALS) as the counter electrode, and a Hg/HgO electrode (ALS, 1 M NaOH internal solution) as the reference electrode. The working electrode was prepared by drop-casting a DI water solution containing 0.00393 mg of nanocrystals onto the glassy carbon surface, resulting in a catalyst loading of 0.02 mg cm^{-2} after overnight drying. Prior to electrocatalytic evaluation, the electrode surface was conditioned by performing 150 cyclic voltammetry (CV) cycles at a scan rate of 500 mV s^{-1} in Ar-saturated 1 M KOH electrolyte within the potential range of 0.05 to 1.05 V versus the reversible hydrogen electrode (RHE). For EOR measurements, CV curves were recorded in Ar-saturated 1 M ethanol + 1 M KOH solution at a scan rate of 50 mV s^{-1} within the same potential range. To ensure fair comparison across cycles (50th, 150th, 250th, 500th), the electrodes and cell were rinsed with DI water, and the electrolyte was replaced with a fresh solution before each specified cycle. Catalytic durability was assessed through chronoamperometric (CA) tests performed at a constant potential of 0.5 V (vs. RHE) for three consecutive runs of 5500 s each, with two CVs recorded between each run. Determination of the electrochemically active surface area (ECSA) for HEA catalysts requires careful consideration due to the presence of multiple surface elements. To ensure reliability, ECSA was evaluated using two independent methods, double-layer capacitance (C_{dl}) and CO stripping, and the results were cross-compared. C_{dl} was measured using CV at scan rates of 25, 50, 100, 150, 200, 300, 400, and 500 mV s^{-1} in Ar-saturated 1 M KOH electrolyte [48]. The potential window for C_{dl} measurements was 100 mV, centered at the open circuit potential, ensuring a non-faradaic process. The double-layer capacitance was calculated from the slope of the plot of cathodic and anodic current versus scan rate [18, 49]. The specific activity was then evaluated using $I/C_{dl} = A\ F^{-1}$, where A and F denote amperes and farads, respectively. CO stripping measurements for ECSA determination were conducted in 1 M KOH following the 1st, 50th, and 250th EOR cycles. After each stage, the electrolyte was refreshed and the electrode was thoroughly rinsed to ensure consistent conditions. CO adsorption was achieved by purging high-purity CO into the electrolyte for 20 min, followed by holding the electrode at 0.1 V (vs RHE) for 10 min. Residual CO in solution was subsequently removed by purging N_2 for 20 min. CO stripping voltammograms were then recorded between 0.05 and 1.2 V (vs. RHE) at a scan

rate of 50 mV s^{-1} for two cycles. The ECSA was calculated by integrating the CO oxidation charge and assuming a conversion factor of 420 μC cm^{-2} for a monolayer of adsorbed CO [50]. The EOR current measured immediately before each CO stripping experiment was then normalized using the corresponding CO-stripping-derived ECSA.

CO Tolerance Evaluation: The experimental setup and electrode preconditioning procedures for CO tolerance experiments were identical to those described in the *Electrocatalytic Measurements for EOR* section. Prior to each CO tolerance measurement, the electrolyte was saturated with CO by purging at a flow rate of 20 cm^3 min^{-1} for 30 min. For prolonged tolerance tests, CV curves were recorded in the same potential region as the surface conditioning process, at a scan rate of 50 mV s^{-1}, while purging of CO. For specific investigations of the 1st, 50th, 150th, and 250th cycles, CV curves were recorded at a scan rate of 20 mV s^{-1}.

Electrocatalytic Measurements in the Presence of Carbon Species: The electrochemical measurements for probing carbon species were conducted in Ar-saturated 1 M KOH electrolytes, each individually containing 1 M of a specific probe intermediate (e.g., CH_3CHO, HCOOH, CH_3COOH). The experimental setup and electrode preconditioning procedures were identical to those detailed in the *Electrocatalytic Measurements for EOR* section. After the preconditioning process, three CV scans were recorded in the probe-containing electrolyte within the same potential region (vs. RHE) as the EOR test, at a scan rate of 20 mV s^{-1}. Subsequently, the electrolyte was replaced, and the catalyst underwent 250 EOR cycles. Following these cycles, all experimental setups were thoroughly rinsed, and the electrode was re-tested in a fresh probe-containing electrolyte. To prevent cross-contamination, each carbon species was evaluated independently using freshly prepared electrodes.

Materials Characterization: Inductively coupled plasma-optical emission spectrometry (ICP-OES) was performed using a iCAP 7200 Duo (Thermo SCIENTIFIC) to determine elemental compositions. Transmission electron microscopy (TEM) and high-angle annular dark-field scanning transmission electron microscopy (HAADF-STEM) images, along with energy-dispersive X-ray spectroscopy (EDS) mapping, were acquired on a spherical-aberration-corrected JEM-ARM200FTH (JEOL) field emission TEM operated at 200 kV. X-ray diffraction (XRD) patterns for crystal structure analysis were obtained using a D8 (Bruker) X-ray diffractometer. X-ray photoelectron spectroscopy (XPS) was employed to investigate surface chemistry using a PHI QuanteraII (ULVAC-PHI) high-resolution X-ray photoelectron spectrometer.

LEIS of Pd@$Pd_{0.2}Pt_{0.2}Ir_{0.2}Ru_{0.2}Rh_{0.2}$ Core-Shell Nanocrystals Pre-and Post-250 cycles of EOR**:** Low-energy ion scattering (LEIS) was employed to characterize Pd@$Pd_{0.2}Pt_{0.2}Ir_{0.2}Ru_{0.2}Rh_{0.2}$ core-shell nanocrystals before and after electrocatalytic EOR cycling. Two distinct sample sets were prepared: "pre-EOR" and "post-250 cycles of EOR." Both sets involved depositing the nanocubes onto a Sigracet 39 BB carbon paper (~0.5 x 1 cm), with a defined working area of approximately 0.1 cm^2. The "post-250 cycles of EOR" sample was subjected to 250 cycles of EOR testing under the identical conditions previously detailed in the *Electrocatalytic Measurements for EOR* section. In contrast, the "pre-EOR" sample did not undergo any electrochemical processing. Prior to LEIS data acquisition, both sample sets underwent an oxygen plasma treatment at approximately 10^{-5} mbar for 30 min to eliminate adventitious carbon contamination from the surface. Surface analysis was conducted using a commercial Qtac100 (IonTOF) LEIS spectrometer, with data processing performed in IonTOF SurfaceLab 7 software. A series of spectra were acquired over a 4 x 4 mm area utilizing a 5 keV Ne^+ beam, with ion doses of approximately 2 x 10^{12} ions cm^{-2}. It's important to note that the analysis ion beam size was approximately 50 µm, allowing for the observation of an ensemble of particles. To compare the "pre-EOR" and "post-250 cycles of EOR" samples, a "Light to Heavy" ratio was utilized. This ratio was determined by integrating the peak areas of the "Light 4d" elements (Ru, Rh, Pd) and the "Heavy 5d" elements (Ir, Pt) following a two-point linear baseline subtraction.

XPS peak curve fitting**:** XPS peak fitting was performed according to established guidelines to ensure accuracy and consistency [22, 51]. For spin-orbit split doublets, the area ratio of the $3p_{3/2}$ to $3p_{1/2}$ peaks was set to 2:1, the $3d_{5/2}$ to $3d_{3/2}$ ratio was set to 3:2, and the $4f_{7/2}$ to $4f_{5/2}$ peaks was set to 4:3. Furthermore, the full width at half maximum (FWHM) and the Gaussian–Lorentzian (GL) mixing percentage were constrained to be identical for peaks corresponding to the same oxidation state of each element in different spin-orbit splitting doublets. The energy separation (Δ) between separated spin-orbit splitting peaks for the same metal was also ensured to be consistent across the samples. All binding energies were calibrated by setting the adventitious carbon C 1s peak energy to 284.8 eV.

XAS**:** X-ray absorption near-edge structure (XANES) and extended X-ray absorption fine structure (EXAFS) analyses were conducted to investigate the K-edges of Pd, Ru, and Rh, and the L_3-edges of Pt and Ir. Measurements were performed in total-fluorescence-yield mode at the BL01 and BL17C beamlines of the National Synchrotron Radiation Research Center (NSRRC, Taiwan), respectively. Metal foils of Pd, Pt, Ir, Ru, and Rh were utilized for monochromator energy correction. Optimal fitting parameters for EXAFS data were

determined using a least-squares curve fitting methodology, implemented within the Athena and Artemis modules of the IFEFFIT software suite. Fitting was performed over a k-range of 3 to 10 $Å^{-1}$ for Pd and Rh, while for Pt, Ir, and Ru, the k-range was from 3 to 9 $Å^{-1}$. All samples were analyzed with an R-range of 1.0 to 3.0 Å. For chemical state analysis of Pd, Ru, and Rh within the HEA, the K-edge absorption positions were identified using Athena software by locating the inflection points in the pre-edge regions. These edge positions were then compared to those of control samples, including metal foils and corresponding oxides.

***SEIRAS*:** For SEIRAS measurements, a distinct cell configuration was employed compared to that described for bulk electrocatalytic measurements. A multilayered substrate consisting of a Si wafer, coated with TiO_2, then Au, and the nanocrystal catalyst, served as the working electrode. The preparation protocol for the working electrode began with a micromachined Si wafer (IRUBIS), which was first coated with a ~20 nm TiO_2 layer via plasma enhanced atomic layer deposition (PEALD) at 200 °C. The PEALD process was conducted on an Oxford Instruments OpAL reactor modified to include a Meaglow 50 series hollow cathode RF plasma source. Deposition of TiO_2 was carried out using 99.999% titanium isopropoxide (TTIP) from Sigma-Aldrich as the titanium source and an O_2/Ar plasma with 60W RF input power at 13.56 MHz. Films were deposited using 400 ALD cycles, and the resulting thickness was checked by ellipsometry on Si(100) witness samples. This PEALD-grown TiO_2 layer ensured a pinhole-free protective barrier, safeguarding the Si wafer from corrosive attack by OH^- ions. Subsequently, an 18 nm Au layer was deposited by vacuum evaporation at a rate of 0.04-0.05 nm s^{-1} under approximately 10^{-6} mbar, as developed in our previous works [30, 52, 53]. Following Au deposition, the metal nanocrystal catalyst layer was applied by hand-spraying a water-ethanol (1:9) solution of nanocrystals onto a 1 x 1 cm region, achieving a catalyst loading of 0.05 mg metal cm^{-2}. The multilayered coated micromachined Si wafer was then assembled into a polyether ether ketone cell body, forming the bottom part of the electrochemical cell, with the Au layer making contact with current collectors. An Ag wire functioned as a pseudo-reference electrode, and a Pt coil served as the counter electrode. The potential of the pseudo-reference electrode was calibrated against the Hg/HgO electrode ($E_{Ag\ wire} = E_{Hg/HgO} + 35$ mV). Prior to SEIRAS testing, the working electrode underwent a conditioning process involving 150 CV cycles from 0.05 to 1.05 V (vs. RHE) at a high scan rate of 500 mV^{-1} in Ar-saturated 1 M KOH electrolyte. The electrolyte was then replaced with 1.5 mL of a solution containing 1 M ethanol + 1 M KOH. After all cell components were assembled, the headspace was purged with Ar for 30 min. The assembled cell was then positioned in the sample compartment of a VERTEX 70v Fourier transform infrared

spectrometer (Bruker), equipped with an MCT (mercury cadmium telluride) detector, and unpolarized IR radiation was used. After connecting the electrodes to the cables, the spectrometer was sealed and evacuated for 30 min, and one cycle of CV scan recorded at a scan rate of 50 mV s^{-1} from 0.05 to 1.05 V (vs. RHE) was performed before collecting the first spectra. This preliminary CV scan prior to SEIRAS data acquisition is to ensure relatively consistent surface conditions for comparative SEIRAS analysis throughout later EOR cycles. Spectra were recorded over approximately 70 seconds, each consisting of 70 coadded interferograms at a spectral resolution of 2 cm^{-1}. A SP50 potentiostat (BioLogic) was used to apply constant potentials in a stepwise manner, with increments or decrements of 0.05V, holding at each potential step for 2 min. Each infrared spectrum was acquired 30 s after a potential change. The spectrum collected at the open circuit potential (OCP) was used as the background spectrum. The reported spectra were plotted as the change in infrared signals relative to the signals obtained at the first forward step of 0.1 V (vs.RHE). For higher EOR cycles, CVs were recorded at a scan rate of 50 mV s^{-1} from 0.05 to 1.05 V (vs. RHE) before collecting the spectra. The PCMW2D correlation maps were calculated using the Python code developed by Prof. Shigeaki Morita (Osaka Electro-Communication University, https://github.com/shigemorita/2Dpy), with a moving window size of 2m + 1 = 5. In the resulting contour plots, warm colors (red to pink) indicate positive correlation intensities, while cool colors (blue to arctic blue) represent negative correlation intensities. PCMW2D of spectroelectrochemical data is particularly useful to identify specific potential windows where a series of related spectral changes occur under dynamic electrochemical conditions. Assuming a linear perturbation, the synchronous PCMW2D analysis represents the gradient of the spectral variations, with red and blue bands to represent positive and negative correlations in absorption changes along the potential window, respectively.

***Computational details**:* Machine-learning interatomic potential (MLIP) calculations were conducted using the Universal Models for Atoms (UMA) framework, a pretrained atomistic foundation model for predicting total energies and atomic forces across diverse chemical environments. The uma-m-1p1 checkpoint was adopted in this work with the OC20 catalyst-domain setting for surface-adsorbate systems. Geometry optimizations were performed until the residual forces on all atoms were less than 0.02 eV $Å^{-1}$. UMA was used to optimize adsorption structures and to calculate MLIP-based adsorption energies for comparison with density functional theory (DFT) results, thereby enabling efficient evaluation of adsorption trends across different surface configurations [54]. All DFT calculations were performed using the Vienna *Ab initio* Simulation Package (VASP) [55, 56]. The exchange-correlation

interactions were described by the revised Perdew–Burke–Ernzerhof (RPBE) functional within the generalized gradient approximation (GGA) [57]. Core-valence interactions were treated using the projector augmented-wave (PAW) method, and the plane-wave energy cutoff was set at 520 eV [58]. Fermi-surface broadening in the metallic HEA systems was treated using the first-order Methfessel–Paxton smearing scheme with a smearing width of 0.20 eV. The Brillouin zone was sampled using a 4 × 5 × 1 Monkhorst–Pack *k*-point grid [59]. Geometry optimizations were performed until the residual forces on all atoms were less than 0.02 eV $Å^{-1}$. Electronic structure analyses were carried out using the VASPKIT post-processing package [60].

To represent the equiatomic $Pd_{0.2}Pt_{0.2}Ir_{0.2}Ru_{0.2}Rh_{0.2}$ surface, 50 special quasi-random structure (SQS) slabs with a (5 × 4), three atomic layers were generated for each of the {100}, {110}, and {111} facets [61, 62]. For each facet, the three slabs exhibiting the highest degree of atomic mixing were selected for further calculations. The lattice constant of the HEA slab was derived from the optimized bulk structures of the constituent metals (Pd, Pt, Ir, Ru, and Rh). Specifically, the lattice constant of each metal was converted into the corresponding metallic radius, and the averaged metallic radius (r_{ave}) was used to define the effective lattice constant of the HEA slab. The adsorption energy was defined as E_{ads}=$E_{slab+ads}$-E_{slab}-$E_{adsorbate}$. Specifically, we computed all accessible top-top bidentate adsorption sites for $HCOO_{ad}$ on the slabs and evaluated their adsorption energies:

$$\Delta E_{ads\ (bi\text{-}HCOO)}=E_{bi\text{-}HCOO_{ad}/slab}-E_{slab}-E_{HCOOH}+\frac{1}{2}E_{H_2}$$

while the enthalpic cost for releasing one O-metal bond to generate a monodentate-like $HCOO_{ad}$ configuration was defined as

$$\Delta E_{ads\ (bi\ to\ mono\text{-}like)}=E_{mono\text{-}like\text{-}HCOO_{ad}/slab}-E_{bi\text{-}HCOO_{ad}/slab}$$

This quantity does not represent the full oxidation barrier to CO_2 formation. Rather, it serves as a chemically meaningful descriptor for the ease with which strongly bound bidentate formate can be converted into a less constrained, more reactive mono-like configuration. This interpretation is supported by prior DFT studies showing that adsorbed formate is typically most stable in a bidentate geometry, whereas further decomposition or oxidation generally requires cleavage of one metal-oxygen bond or reorientation toward a monodentate configuration [41−43]. In these expressions, $E_{bi\text{-}HCOO_{ad}/slab}$ and $E_{mono\text{-}like\text{-}HCOO_{ad}/slab}$ denote the total energies of the slab with bidentate and mono-like HCOO adsorbed, respectively, E_{slab} is the total energy of the clean slab, and E_{HCOOH} and E_{H_2} are the total energies of gas-phase HCOOH and H_2, respectively. All energies are given in eV.

Figures

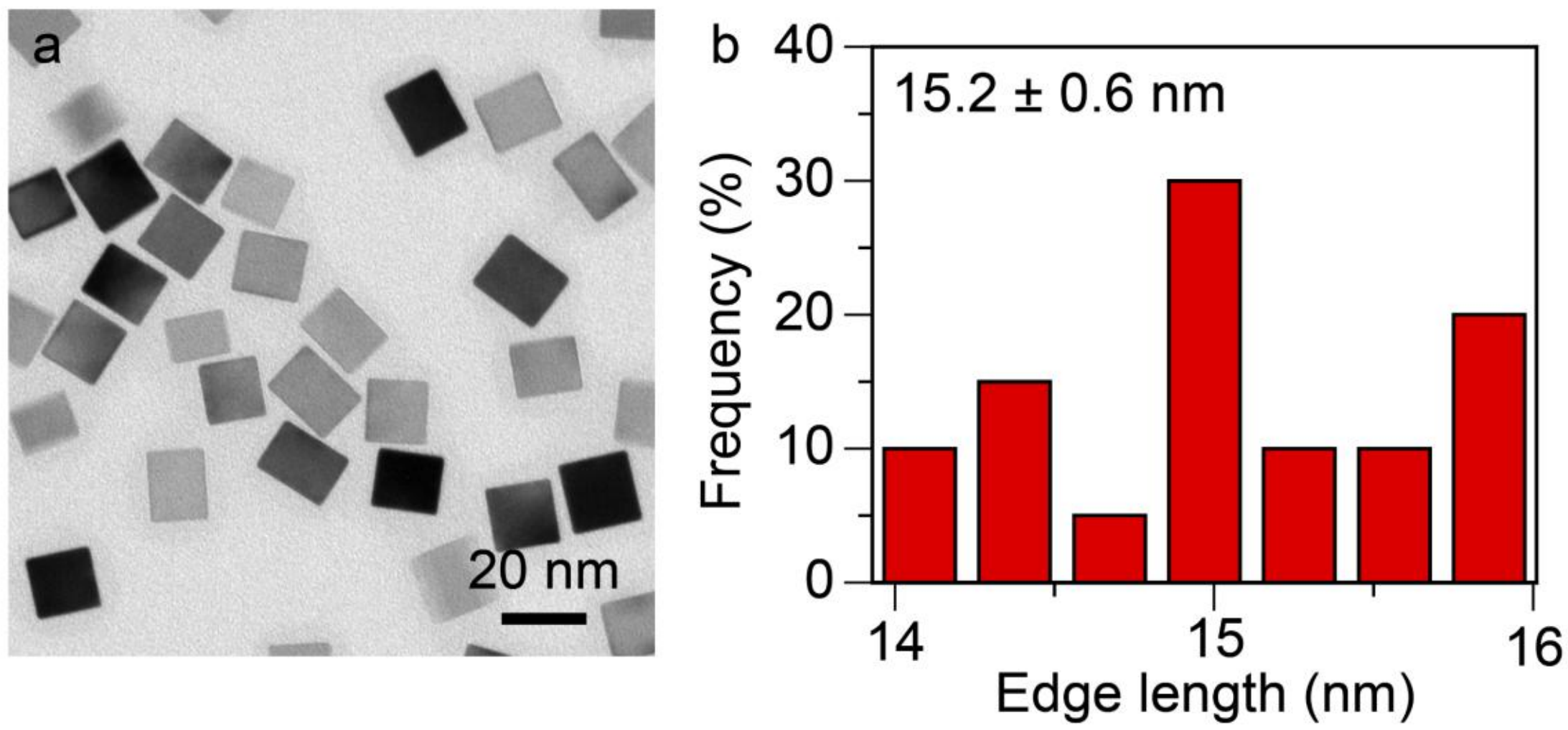

Figure S1. TEM images and size distribution analysis of Pd nanocubes. The particle size distribution was determined from the averaged edge lengths of 20 individual nanocrystals, with the longer edge of each particle used for measurement.

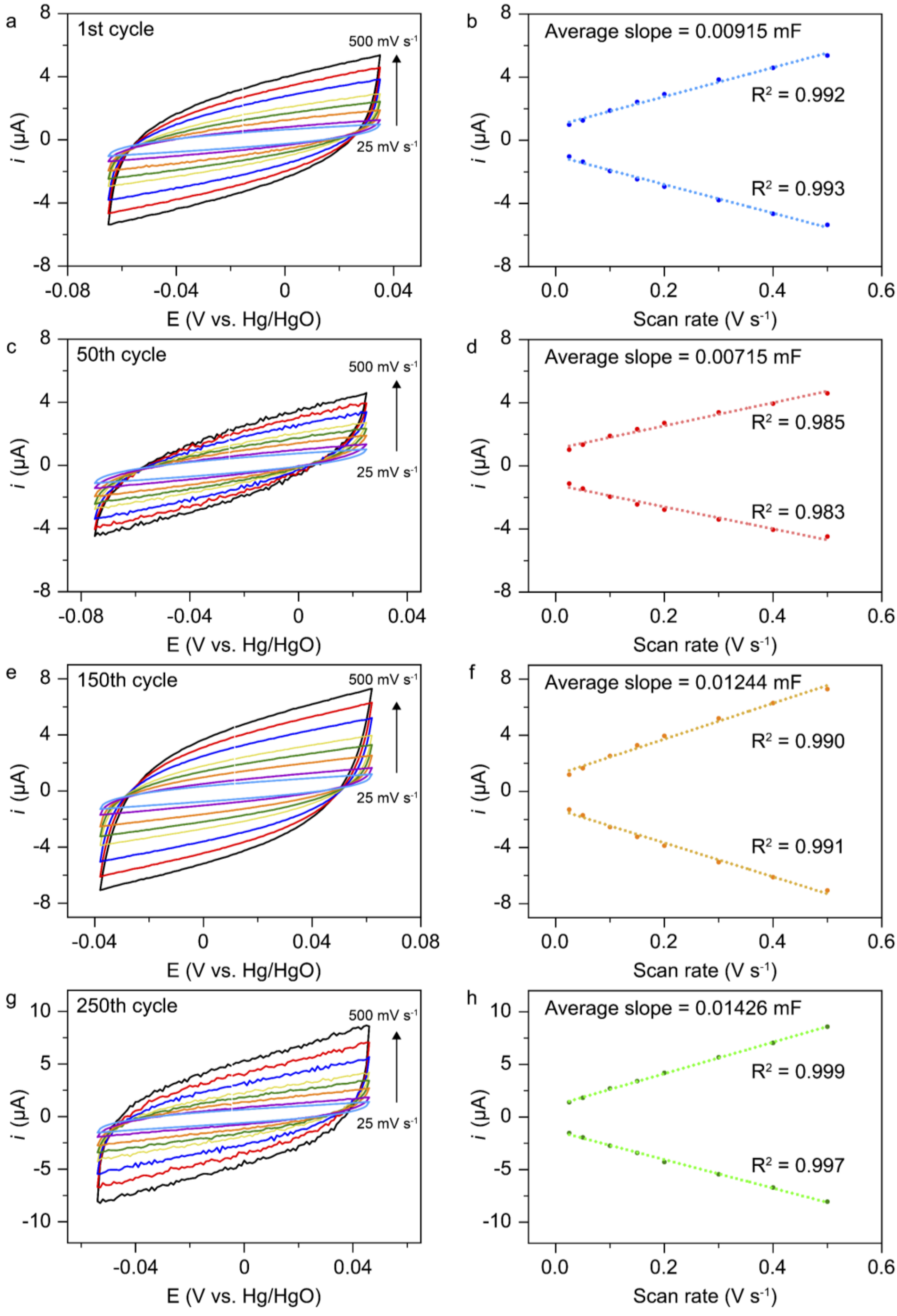

Figure S2. Cyclic voltammograms recorded in Ar-saturated 1 M KOH to determine the double-layer capacitance of $Pd@Pd_{0.2}Pt_{0.2}Ir_{0.2}Ru_{0.2}Rh_{0.2}$ core-shell nanocrystals. Measurements were performed at scan rates ranging from 25 to 500 mV s^{-1}. Panels (a, b) show the 1st, (c, d) 50th,

(e, f) 150th, and (g, h) 250th EOR cycles, with corresponding current versus scan rate plots used to estimate electrochemical double-layer capacitance.

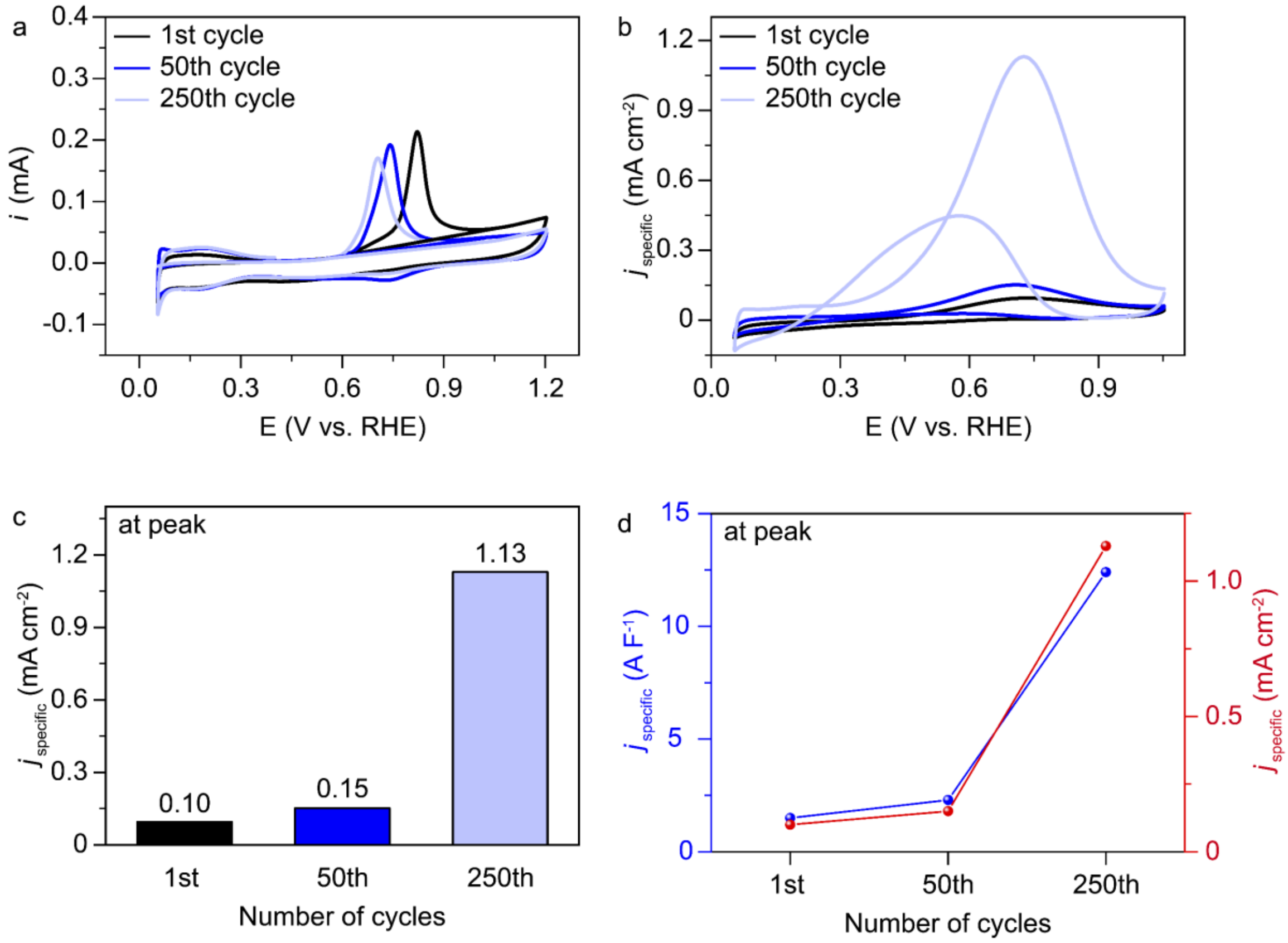

Figure S3. Specific activity of the EOR normalized by the ECSA derived from CO stripping for Pd@$Pd_{0.2}Pt_{0.2}Ir_{0.2}Ru_{0.2}Rh_{0.2}$ core-shell nanocrystals at the 1st, 50th, and 250th EOR cycles. (a) CO stripping curves recorded after the corresponding EOR cycling stages, (b) ECSA-normalized CV profiles for EOR obtained prior to each CO stripping measurement, (c) specific activities extracted from the forward peak current (j_f), and (d) comparison of specific activities normalized using ECSA values derived from CO stripping and from C_{dl}. The close agreement between these two approaches confirms that the C_{dl}-based method used in this work provides a reliable representation of the catalytic performance trends.

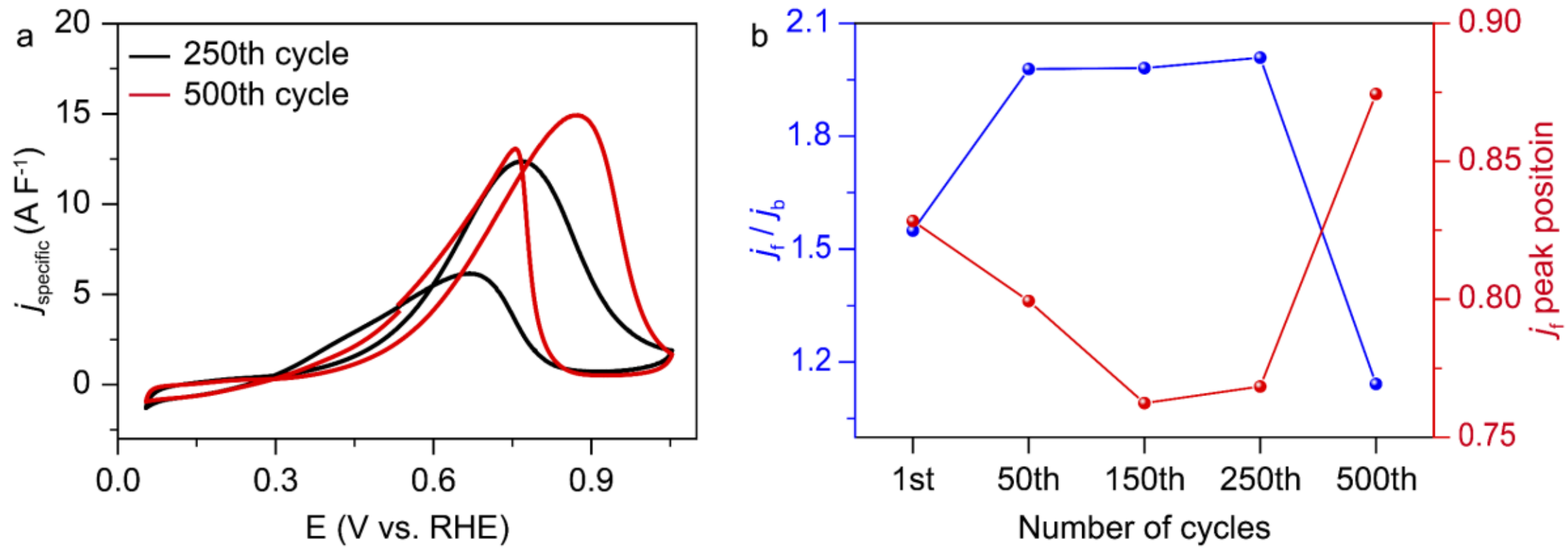

Figure S4. Electrochemical EOR performance of $Pd@Pd_{0.2}Pt_{0.2}Ir_{0.2}Ru_{0.2}Rh_{0.2}$ core-shell nanocrystals at up to the 500th cycle. (a) CV profiles normalized by ECSA, and (b) j_f/j_b ratio and the potential position of the j_f peak.

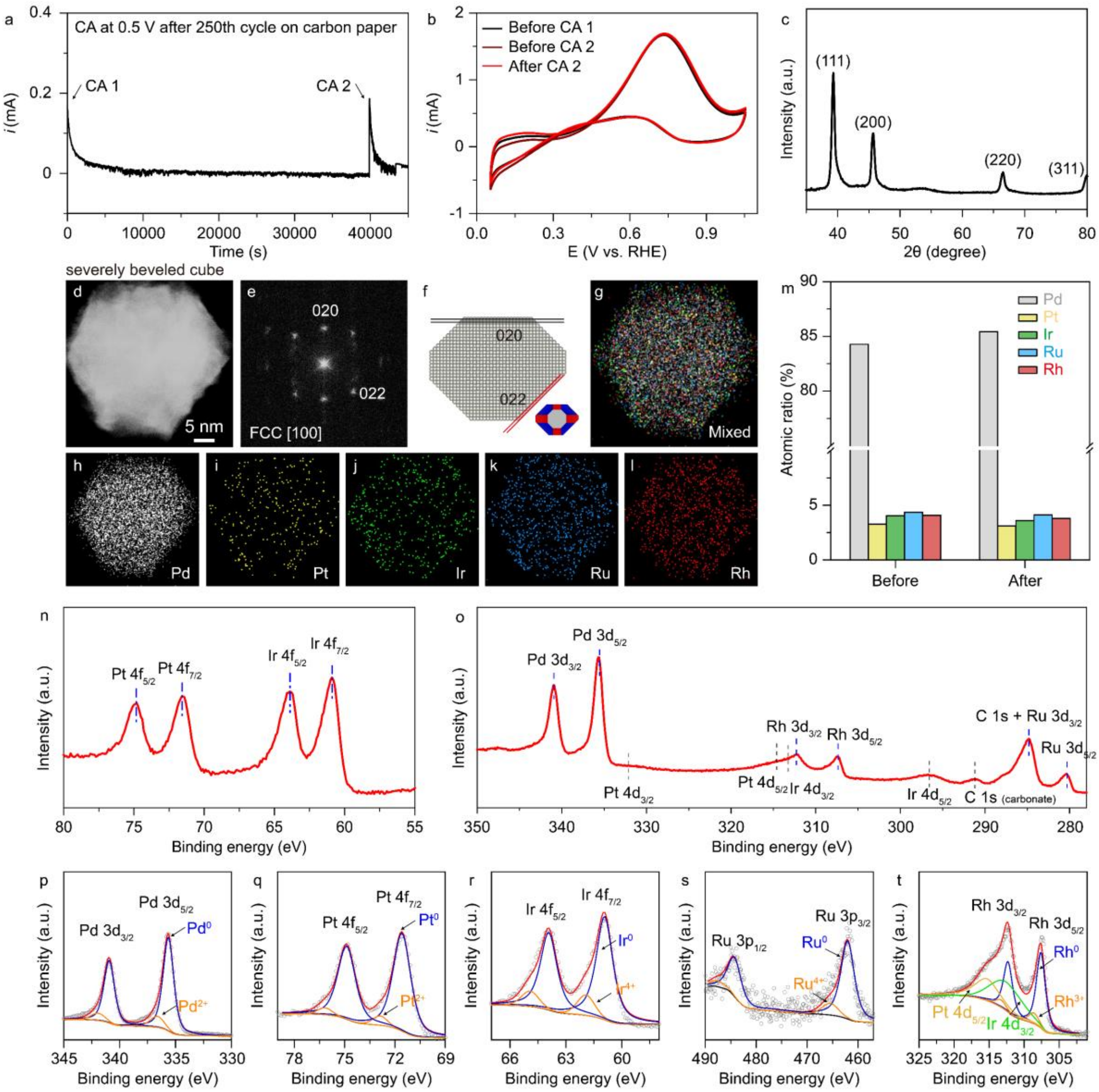

Figure S5. Post-chronoamperometry characterization of $Pd@Pd_{0.2}Pt_{0.2}Ir_{0.2}Ru_{0.2}Rh_{0.2}$ core-shell nanocrystals supported on carbon paper. (a) CA measurements conducted at 0.5 V (vs. RHE) for 40000 + 5000 s following the 250th EOR cycle, with two CV scans recorded between each CA segment, (b) CV profiles corresponding to the 250th EOR cycle, as well as those obtained between and after CA testing. (c) XRD patterns of the catalyst after CA measurements. (d-l) HAADF-STEM images with corresponding (e) FFT patterns, (f) schematic illustrations of facet orientations, together with (g-l) EDS elemental maps. (m) ICP-OES analysis comparing elemental compositions of the pristine (before) and post-CA (after) samples. (n, o) High-resolution XPS survey spectra in the ranges of 55-80 eV and 278-350 eV, respectively. (p-t) Deconvoluted XPS spectra for (p) Pd 3d, (q) Pt 4f, (r) Ir 4f, (s) Ru 3p, and (t) Rh 3d.

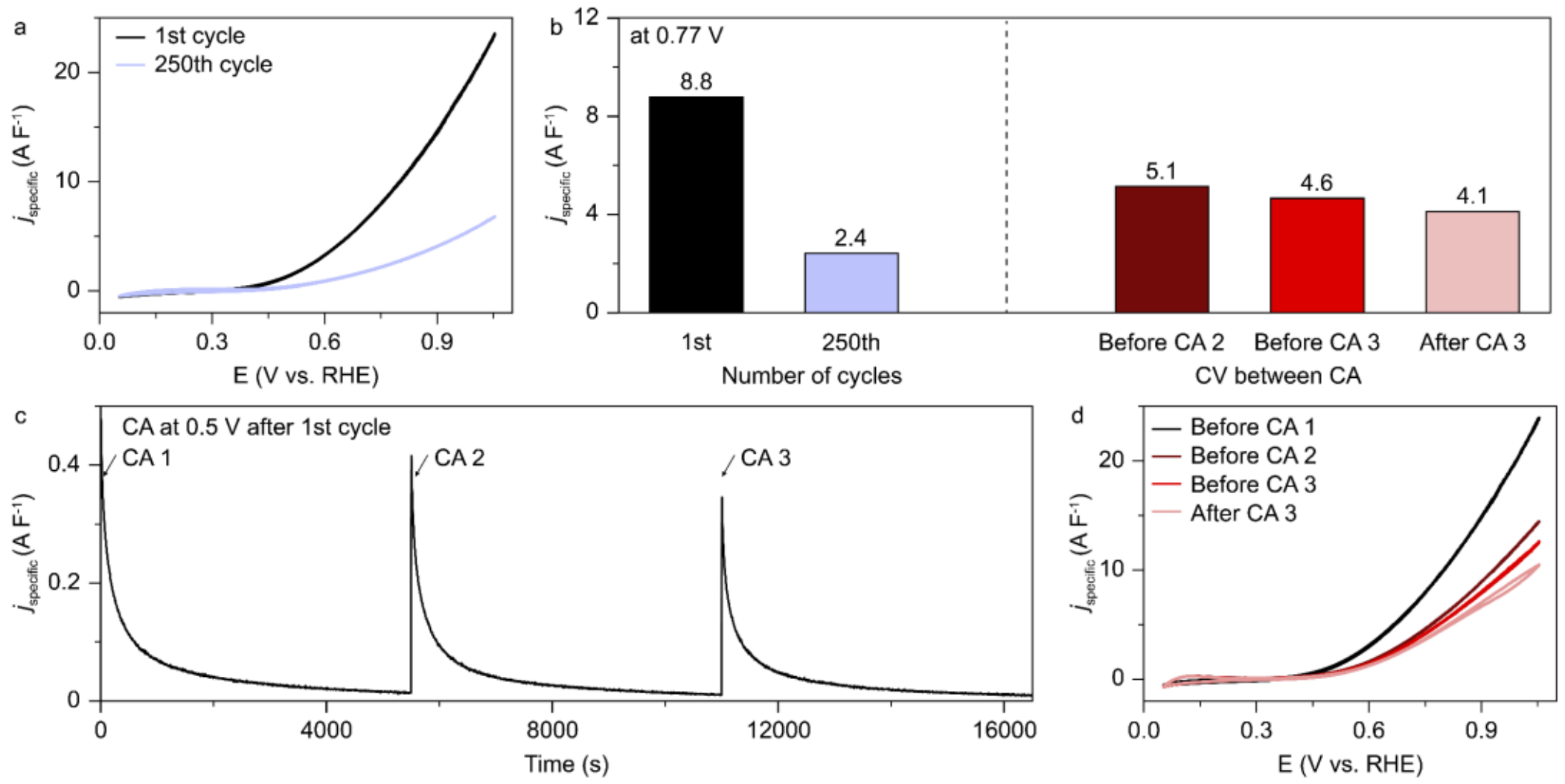

Figure S6. Electrochemical EOR performance of Pd nanocubes. (a) CV normalized by ECSA, measured from 0.05 to 1.05 V vs. RHE at a scan rate of 50 mV·s^{-1} for the 1st and 250th cycles in Ar-saturated 1 M KOH + 1 M ethanol. (b) Specific activities (ECSA-normalized) at 0.77 V in the forward scan: comparison across EOR cycles (left); comparison of EOR profiles shown in the panel (d), recorded before, between, and after CA tests (right). (c) CA tests performed at 0.5 V vs. RHE after the 1st EOR cycle, with two CVs collected between each CA segment. (d) CV profiles for the first EOR cycle, between, and after CA tests.

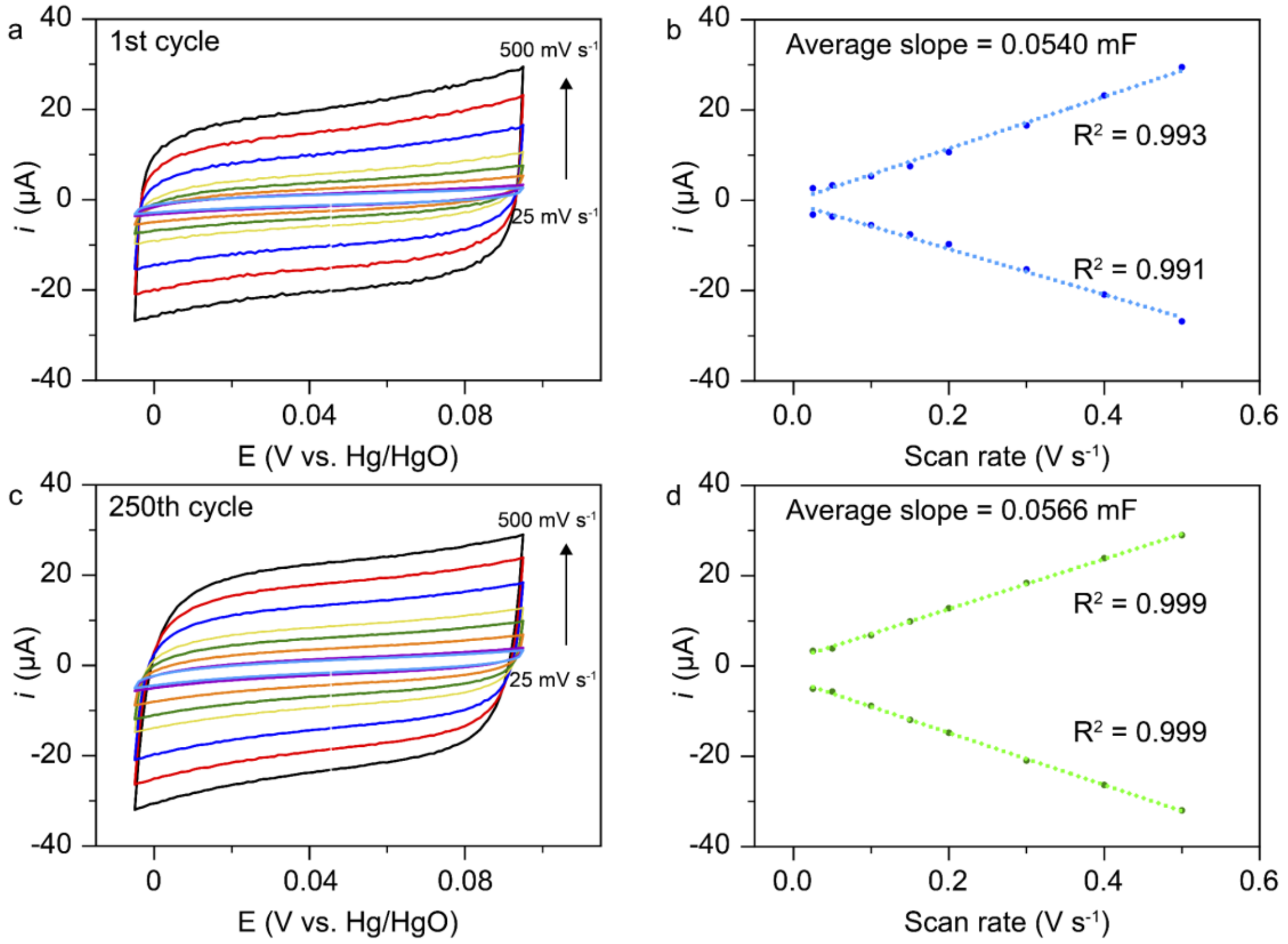

Figure S7. Cyclic voltammograms recorded in Ar-saturated 1 M KOH to determine the double-layer capacitance of Pd nanocubes. Measurements were performed at scan rates ranging from 25 to 500 mV s^{-1}. Panels show (a, b) the 1st, and (c, d) 250th EOR cycles, with corresponding plots of charging/discharging current versus scan rate used to estimate the electrochemical double-layer capacitance.

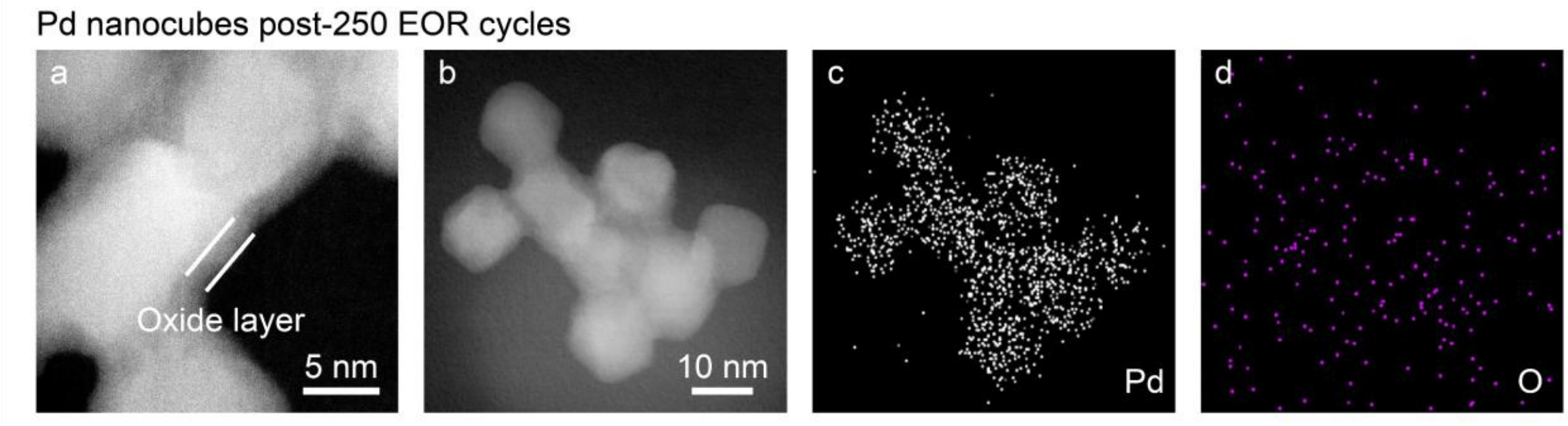

Figure S8. (a, b) HAADF-STEM images of Pd nanocubes after the 250th EOR cycle. (c, d) Corresponding EDS elemental maps of (b), showing the spatial distributions of (c) Pd and (d) O.

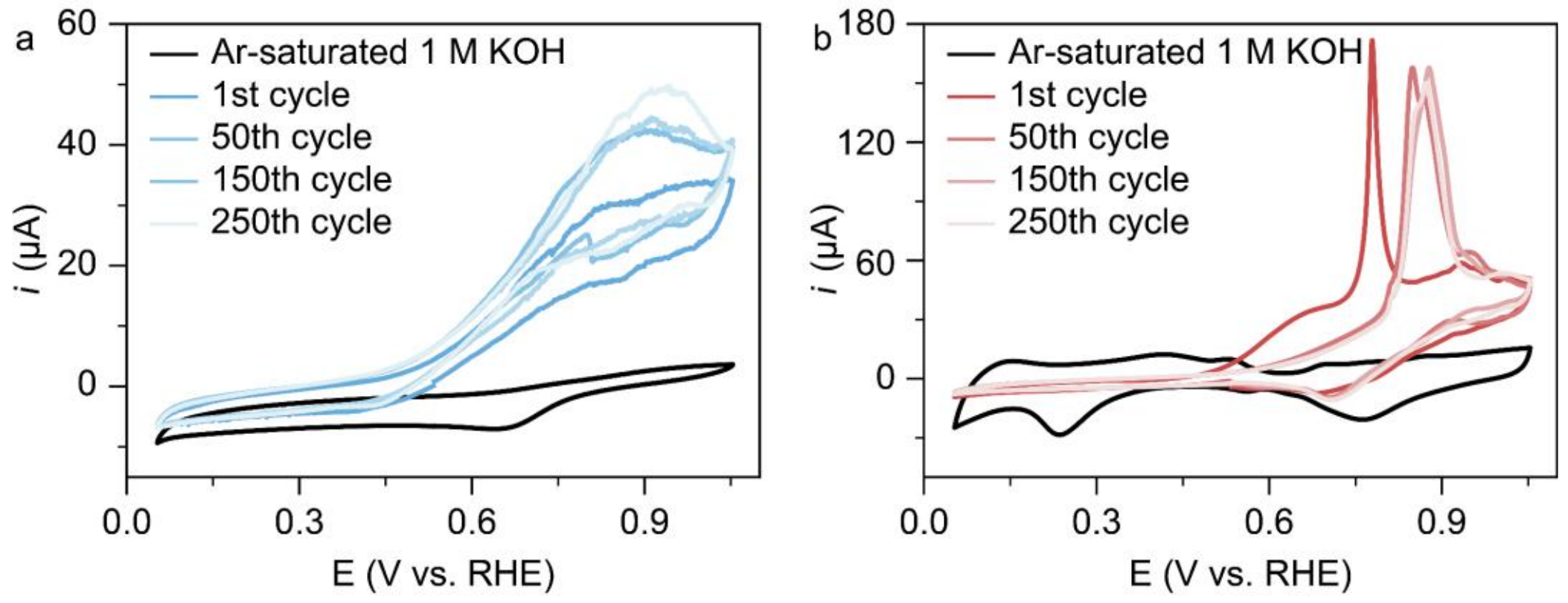

Figure S9. CO tolerance test for (a) $Pd@Pd_{0.2}Pt_{0.2}Ir_{0.2}Ru_{0.2}Rh_{0.2}$ core-shell nanocrystals, and (b) Pd nanocubes. Comparison between the CV recorded in Ar-saturated 1 M KOH (black line) and CO-saturated 1 M KOH (blue and red lines) is shown. CV's recorded with a scan rate of 20 mV s^{-1}.

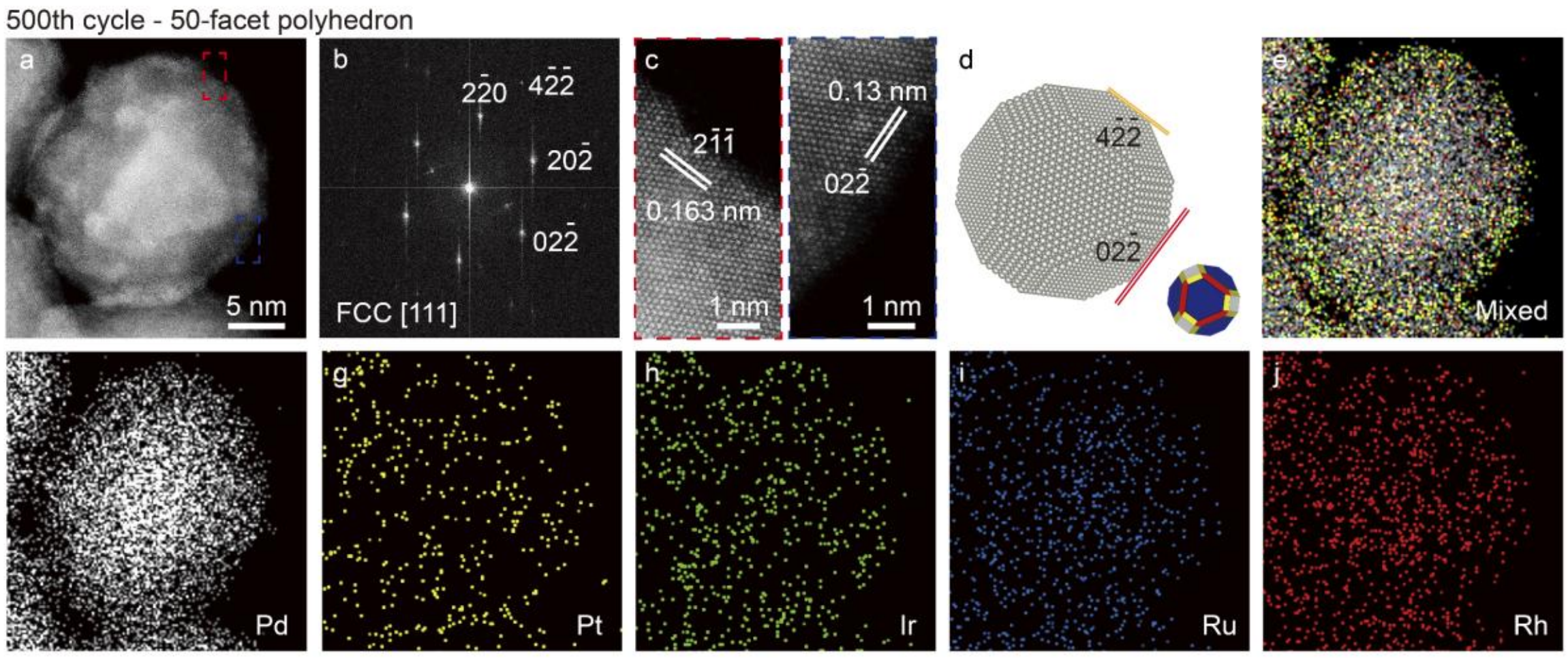

Figure S10. (a, c) HAADF-STEM images, (b) corresponding FFT patterns for (a), (d) schematic facet orientation illustrations, and (e-j) EDS elemental maps showing (e) elemental overlaps, (f) Pd, (g) Pt, (h) Ir, (i) Ru, and (j) Rh for Pd@$Pd_{0.2}Pt_{0.2}Ir_{0.2}Ru_{0.2}Rh_{0.2}$ core-shell nanocrystals after the 500th EOR cycle. The nanocrystals retained a morphology similar to that observed after 250 cycles, albeit slightly more beveled. The particles evolved toward a nearly rhombicuboctahedral geometry, characterized by well-defined 26 facets and a balanced exposure of {100}, {110}, and {111} planes. Elemental mapping and ICP-OES (Table S2) analysis confirm that the elemental distribution remains uniform throughout the structure. Therefore, the slight decline in EOR performance observed at the 500th CV compared to the 250th CV is ascribable to the gradual deactivation of active sites rather than structural degradation or compositional segregation.

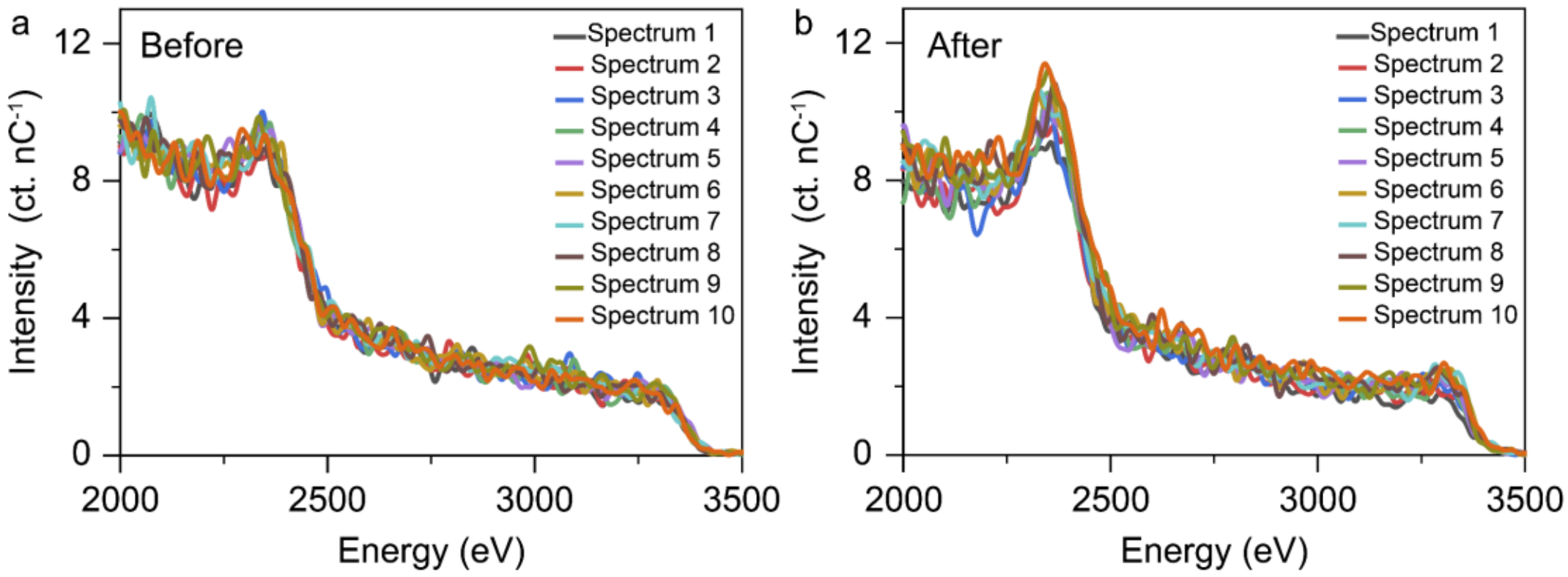

Figure S11. LEIS spectra used for averaging in Figure 3 for $Pd@Pd_{0.2}Pt_{0.2}Ir_{0.2}Ru_{0.2}Rh_{0.2}$ core-shell nanocrystals. (a) pre- and (b) post-EOR (250 cycles).

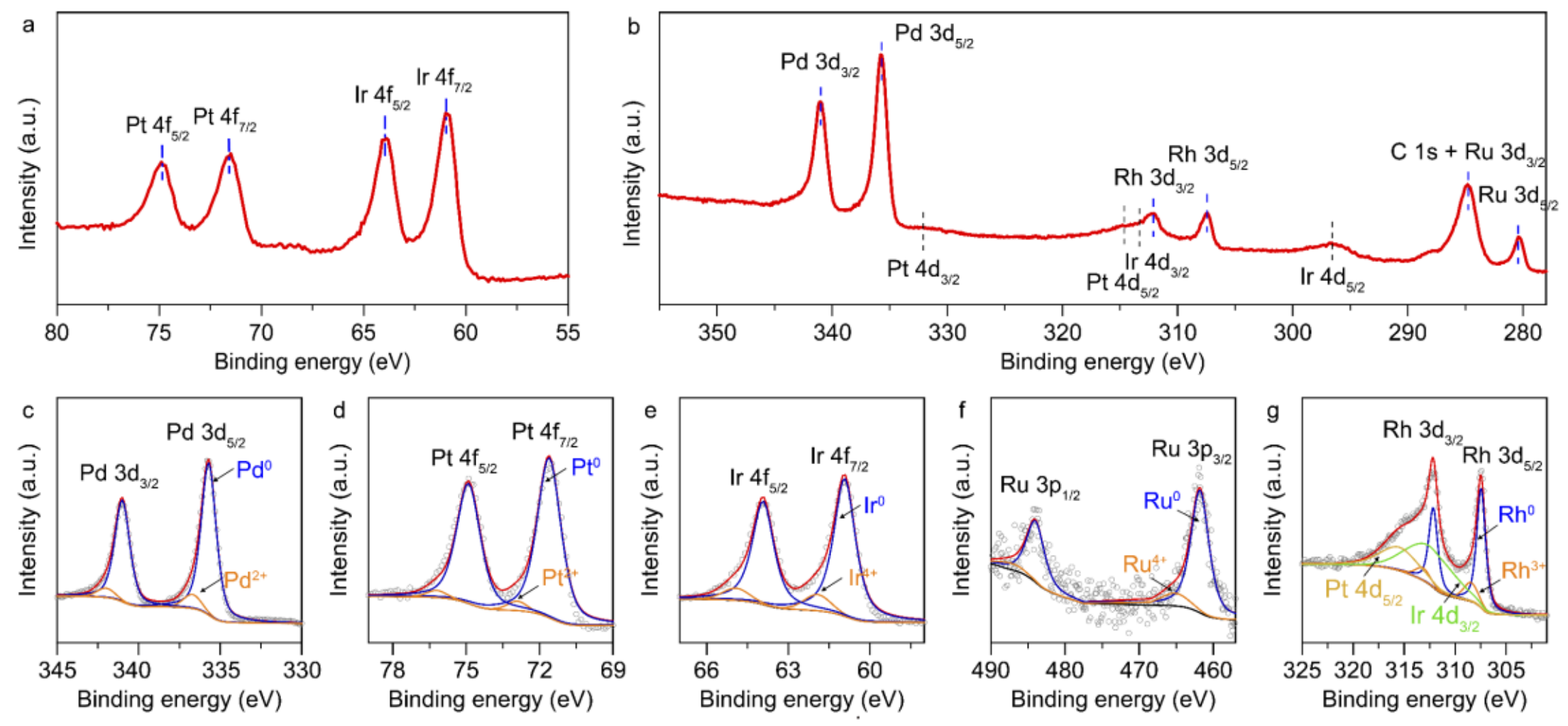

Figure S12. XPS analysis and corresponding peak fitting for $Pd@Pd_{0.2}Pt_{0.2}Ir_{0.2}Ru_{0.2}Rh_{0.2}$ core-shell nanocrystals before electrocatalysis. High-resolution survey scans and elemental regions are shown for (a) 55-80 eV and (b) 278-355 eV. Fitted peaks are presented for (c) Pd 3d, (d) Pt 4f, (e) Ir 4f, (f) Ru 3p, and (g) Rh 3d.

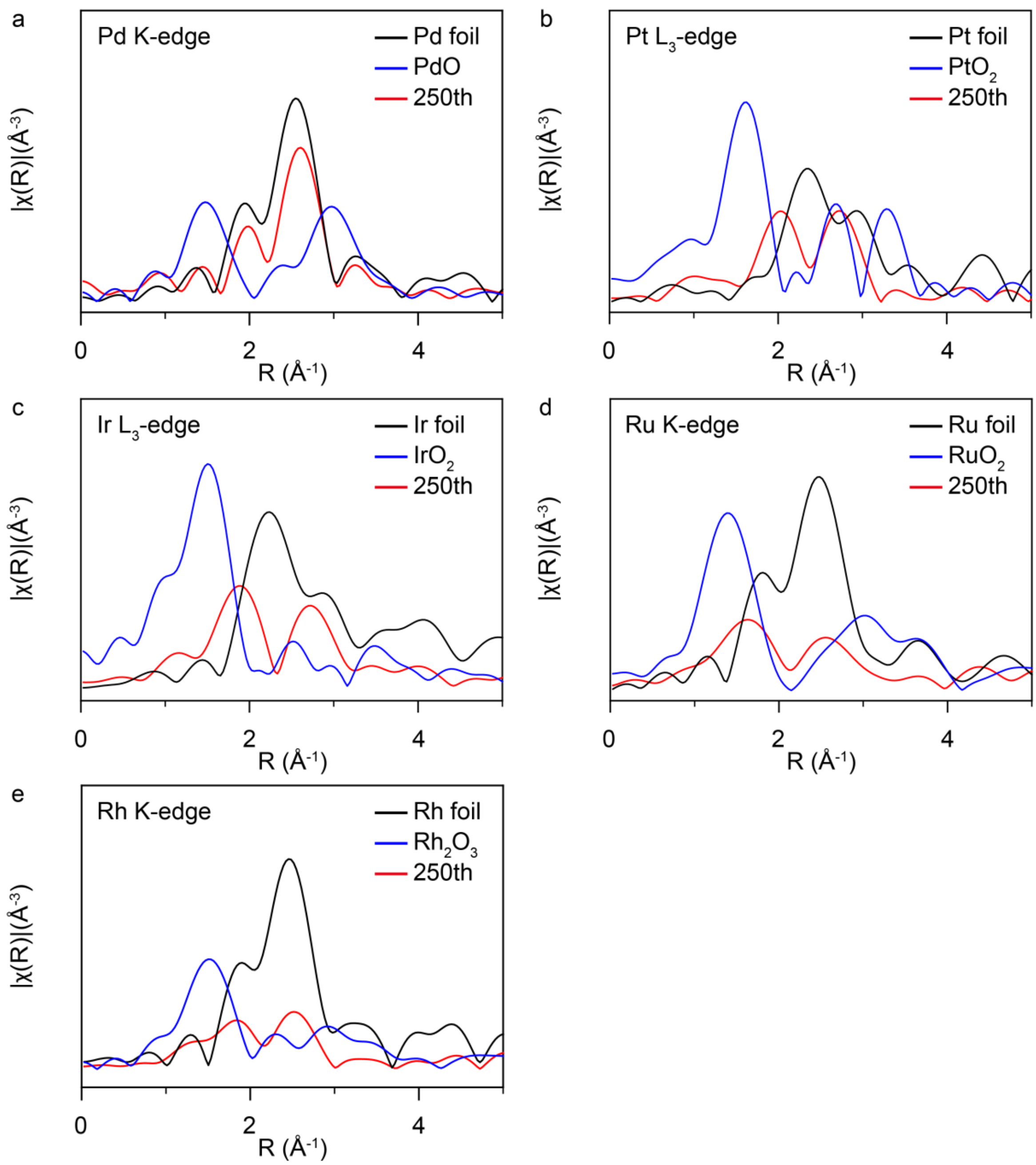

Figure S13. FT-EXAFS spectra of $Pd@Pd_{0.2}Pt_{0.2}Ir_{0.2}Ru_{0.2}Rh_{0.2}$ core-shell nanocrystals obtained after 250 EOR cycles, compared with their corresponding metal foils and oxide references. Spectra are shown for (a) Pd K-edge, (b) Pt L_3-edge, (c) Ir L_3-edge, (d) Ru K-edge, and (d) Rh K-edge. The difference between the Ru K-edge XANES spectrum of $Pd@Pd_{0.2}Pt_{0.2}Ir_{0.2}Ru_{0.2}Rh_{0.2}$ after 250 EOR cycles and that of Ru foil can be rationalized by the distinct crystallographic and electronic environments of Ru in the HEA lattice (Figure 3l) [63, 64]. Metallic Ru foil adopts the thermodynamically stable hexagonal close-packed (HCP) structure, whereas Ru atoms in $Pd@Pd_{0.2}Pt_{0.2}Ir_{0.2}Ru_{0.2}Rh_{0.2}$ are incorporated into a FCC-like multimetallic lattice dictated by the Pd core and the overall HEA framework. The HCP-to-FCC structural difference, together with strong Ru-Pd/Pt/Ir/Rh electronic coupling, can substantially

modify the Ru local density of states and multiple-scattering features. In particular, the enhanced white-line suggests an increased density of unoccupied Ru-centered states with mixed 4d/5p character, arising from alloy-induced d-d orbital hybridization and local symmetry modification. Importantly, the Ru K-edge position remains close to that of Ru foil and is clearly distinct from oxidized Ru references, confirming that Ru is predominantly retained in a metallic/reduced state after EOR cycling. Therefore, the modified Ru XANES line shape should be attributed mainly to the FCC HEA coordination environment and multimetallic electronic hybridization, rather than to substantial Ru oxidation.

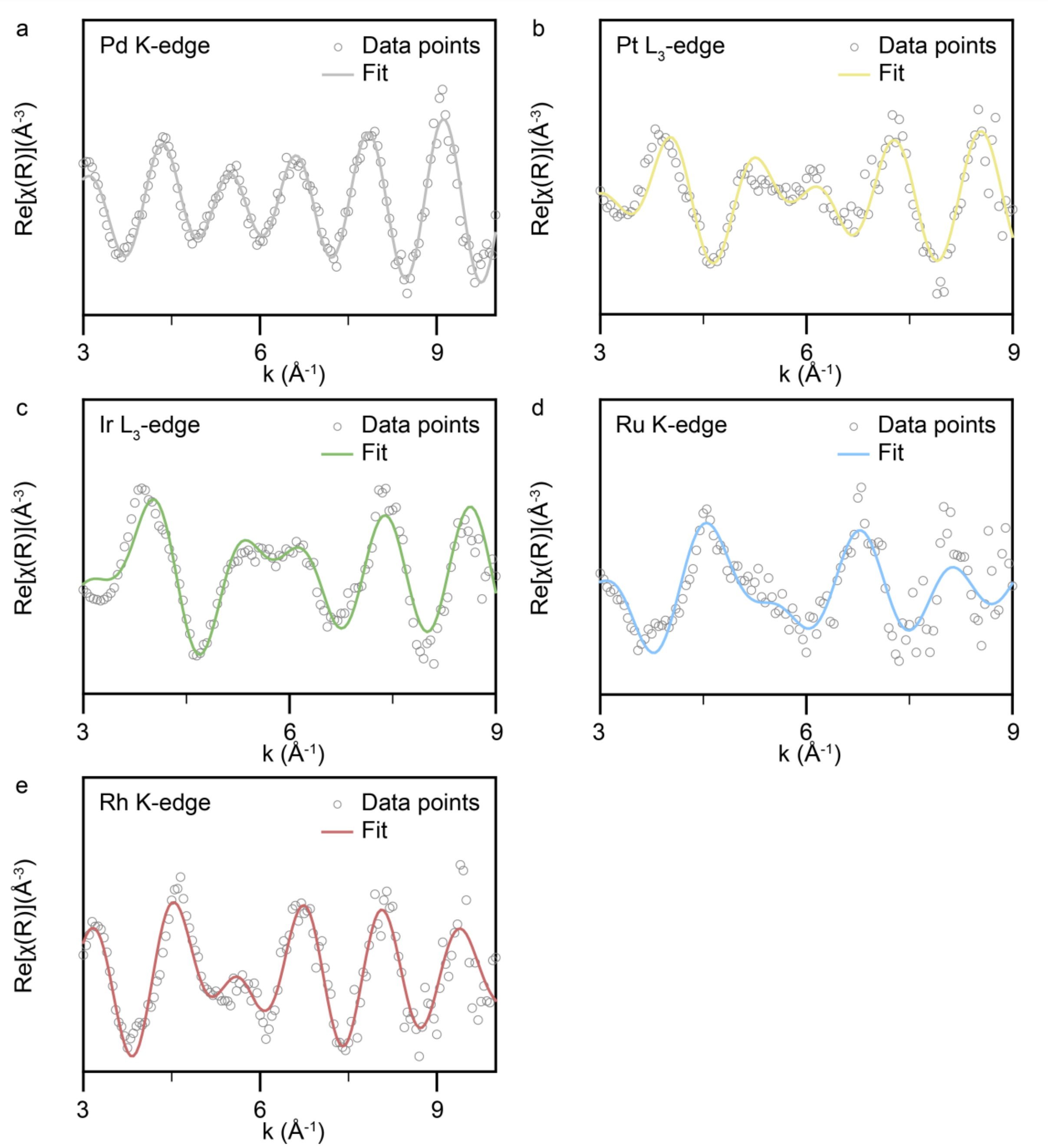

Figure S14. FT-EXAFS spectra in k space for $Pd@Pd_{0.2}Pt_{0.2}Ir_{0.2}Ru_{0.2}Rh_{0.2}$ core-shell nanocrystals after 250 EOR cycles, along with the corresponding fitting curves. Spectra are presented for (a) Pd K-edge, (b) Pt L_3-edge, (c) Ir L_3-edge, (d) Ru K-edge, and (d) Rh K-edge.

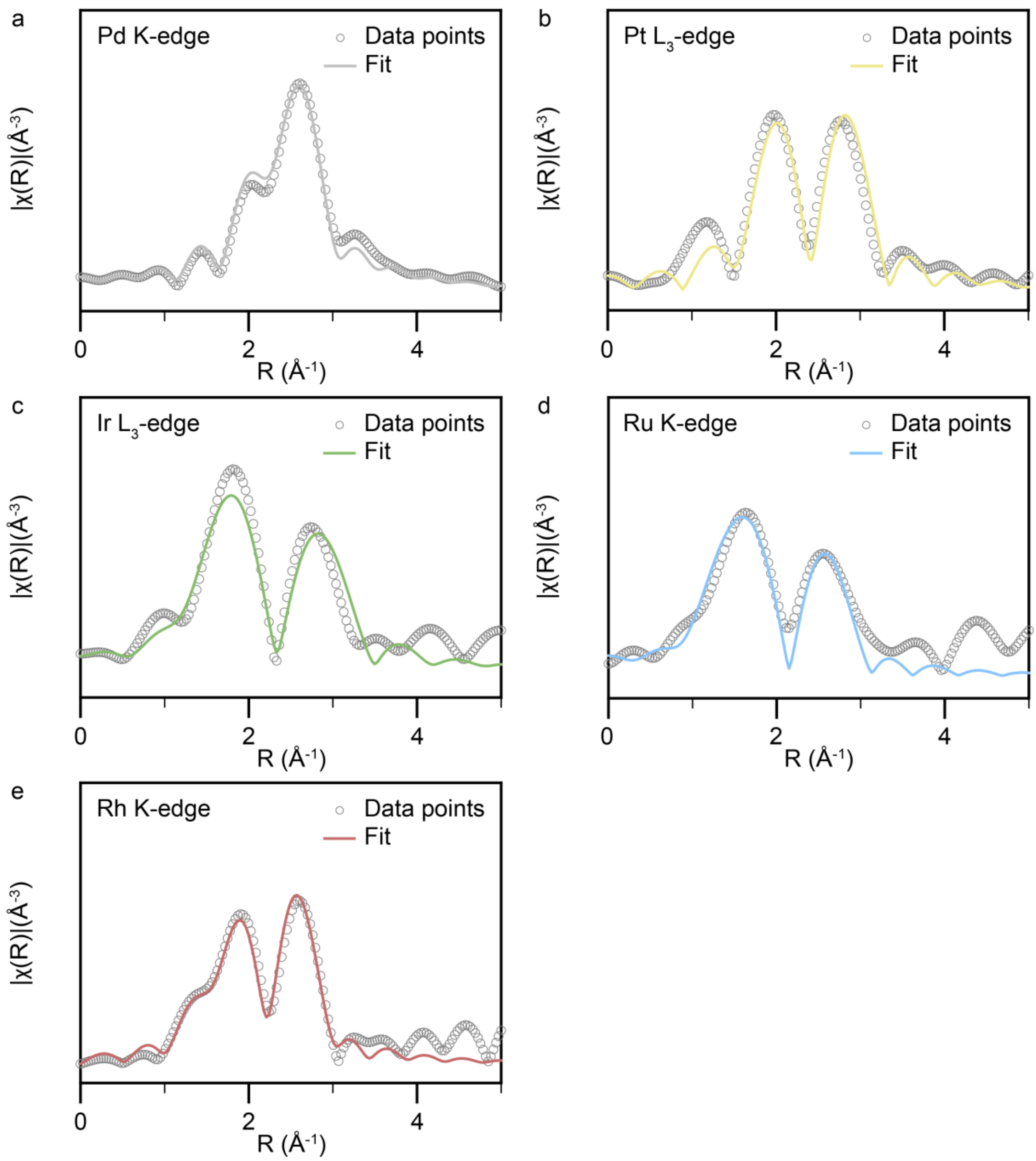

Figure S15. FT-EXAFS spectra in R space for Pd@$Pd_{0.2}Pt_{0.2}Ir_{0.2}Ru_{0.2}Rh_{0.2}$ core-shell nanocrystals after 250 EOR cycles, along with the corresponding fitting curves. Spectra are presented for (a) Pd K-edge, (b) Pt L_3-edge, (c) Ir L_3-edge, (d) Ru K-edge, and (d) Rh K-edge.

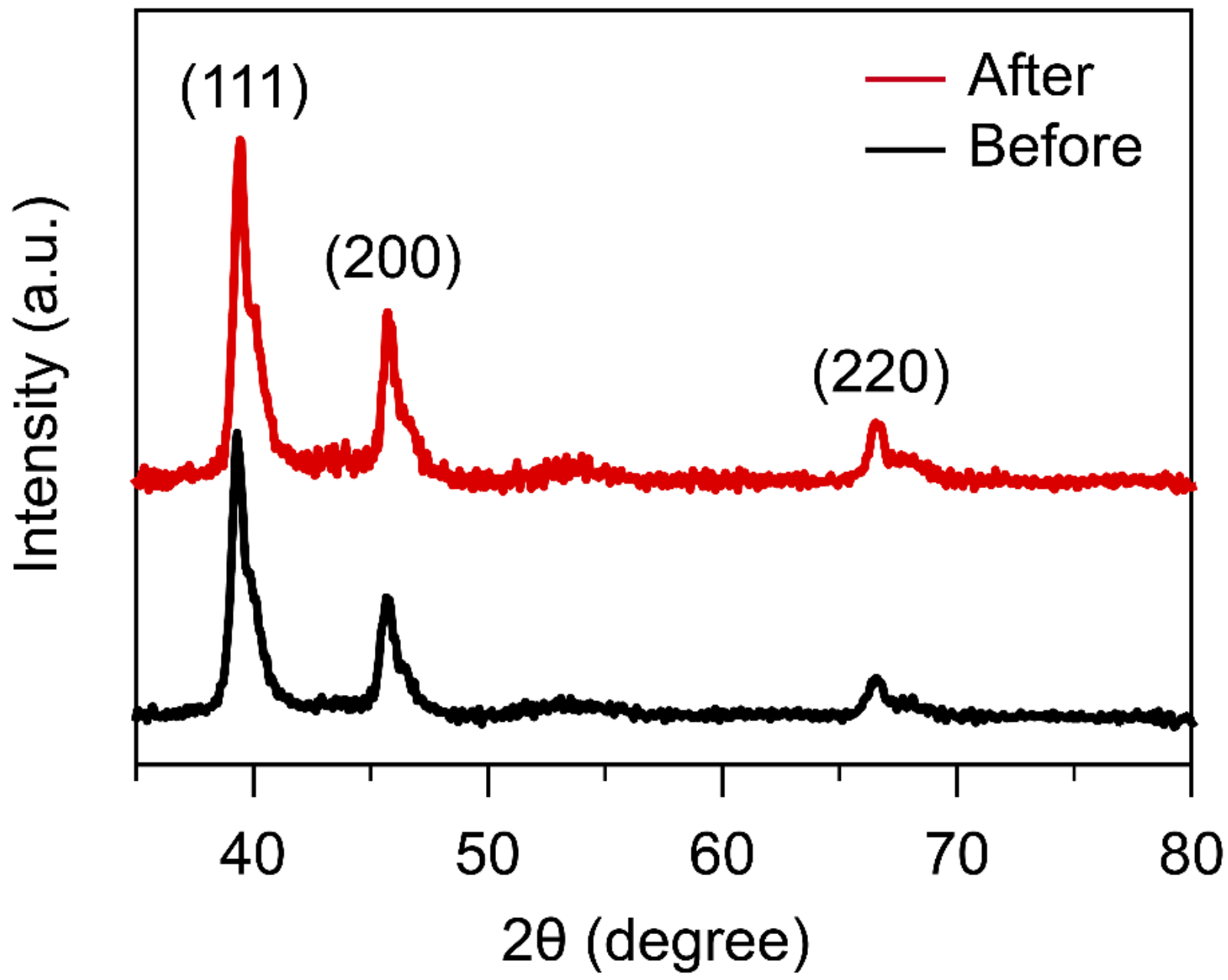

Figure S16. XRD diffraction patterns for $Pd@Pd_{0.2}Pt_{0.2}Ir_{0.2}Ru_{0.2}Rh_{0.2}$ core-shell nanocrystals before and after 250 EOR cycles. The diffraction peaks appear at $2\theta = 39.3°$, 45.7°, and 66.5° in the pristine sample, corresponding to the (111), (200), and (220) planes, respectively, and remain essentially unchanged after 250 cycles, with peak positions at 39.4°, 45.7°, and 66.5°. No additional reflections associated with phase separation, secondary phases, or oxide formation are observed, indicating that the core-shell nanocrystals preserve their phase uniformity throughout the electrochemical cycling process.

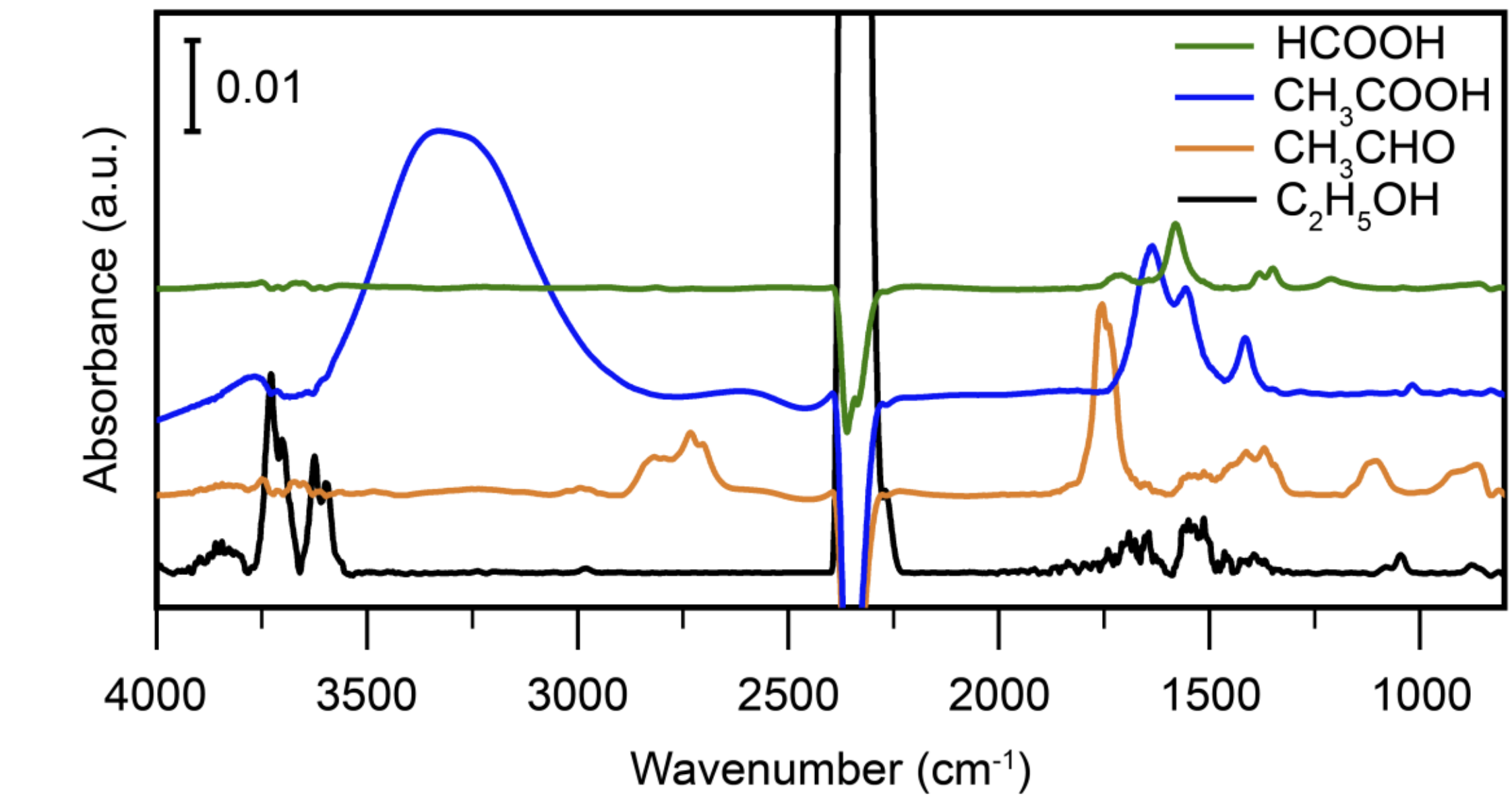

Figure S17. SEIRAS spectra were collected by directly dropping control electrolytes, each containing 1 M of individual products (CH_3COOH, HCOOH, CH_3CHO, and C_2H_5OH) in 1 M KOH, onto the electrode surface, without vacuuming the chamber or assembling the full electrochemical cell.

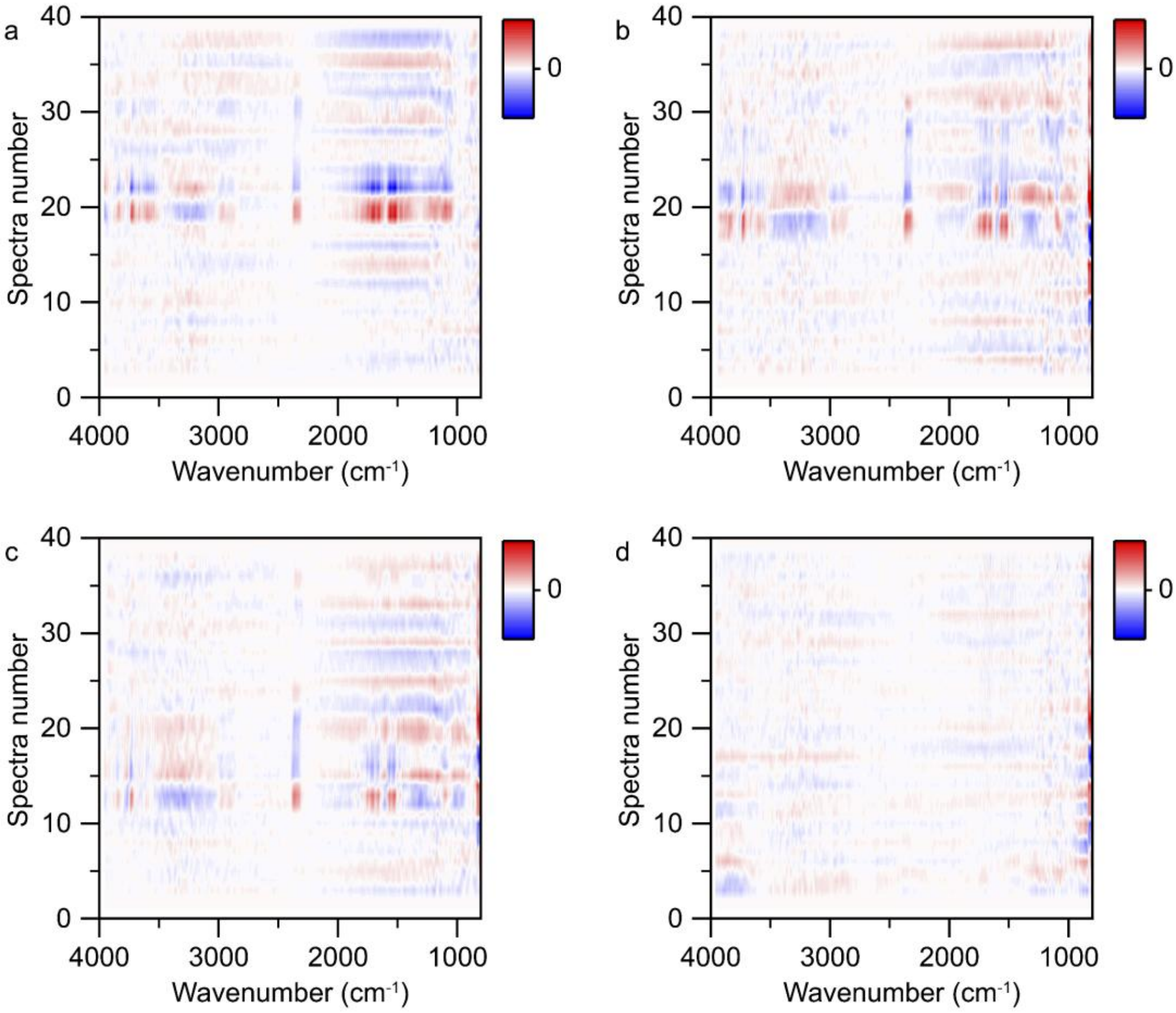

Figure S18. Asynchronous PCMW2D correlation maps highlighting the curvature of band absorption changes during potential sweeps obtained from in situ SEIRAS spectral analysis during the EOR for $Pd@Pd_{0.2}Pt_{0.2}Ir_{0.2}Ru_{0.2}Rh_{0.2}$ core-shell nanocrystals. Panels show results from the (a) 1st, (b) 50th, (c) 150th, and (d) 250th EOR cycles.

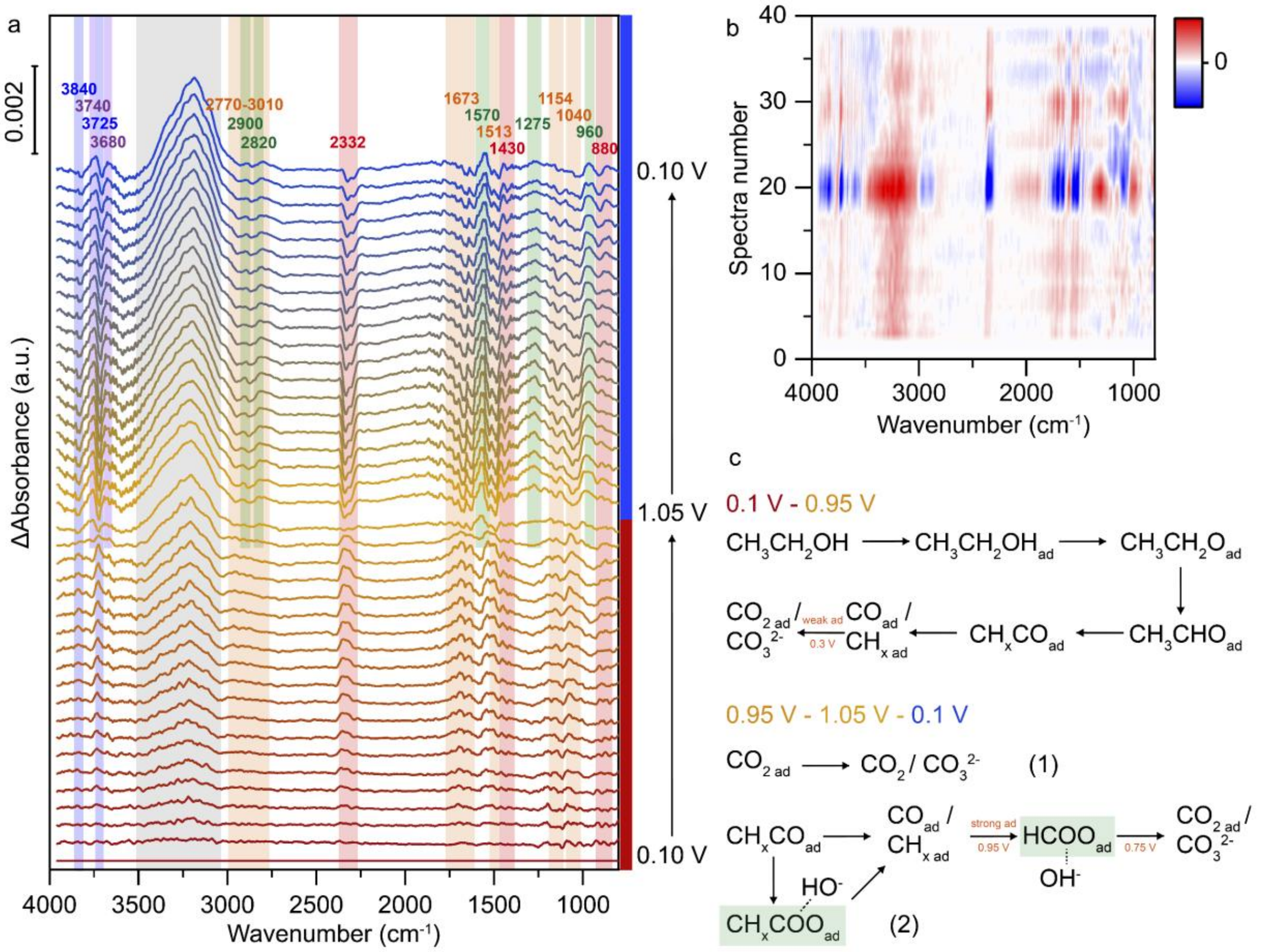

Figure S19. In situ SEIRAS spectral analysis during the 50th EOR cycle for $Pd@Pd_{0.2}Pt_{0.2}Ir_{0.2}Ru_{0.2}Rh_{0.2}$ core-shell nanocrystals in 1 M KOH + 1 M ethanol (Ar-saturated) electrolyte. (a) SEIRAS spectra collected with a potential step of 0.05 V at a potential window of 0.1-1.05 V vs. RHE. (b) Synchronous PCMW2D correlation map highlighting dynamic changes in absorption during potential sweeps. (c) Proposed EOR pathway on $Pd@Pd_{0.2}Pt_{0.2}Ir_{0.2}Ru_{0.2}Rh_{0.2}$ core-shell nanocrystals during the 50th cycle. The band species assignments for the colored boxes in panel (a) are consistent with those shown in Figure 4 and listed in Table S6.

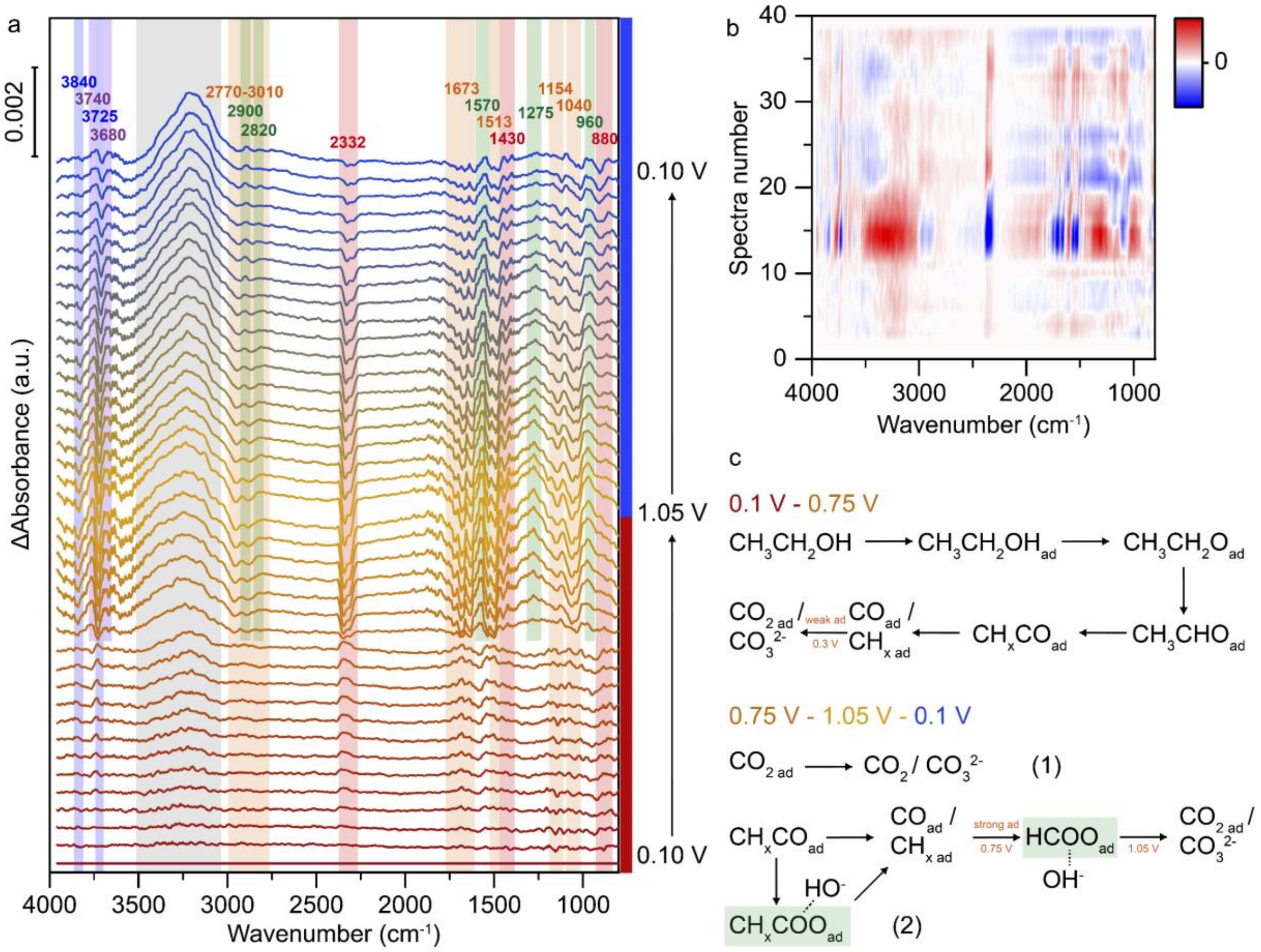

Figure S20. In situ SEIRAS spectral analysis during the 150th EOR cycle for Pd@Pd$_{0.2}$Pt$_{0.2}$Ir$_{0.2}$Ru$_{0.2}$Rh$_{0.2}$ core-shell nanocrystals in 1 M KOH + 1 M ethanol (Ar-saturated) electrolyte. (a) SEIRAS spectra collected with a potential step of 0.05 V at a potential window of 0.1-1.05 V vs. RHE. (b) Synchronous PCMW2D correlation map highlighting dynamic changes in absorption during potential sweeps. (c) Proposed EOR pathway on Pd@Pd$_{0.2}$Pt$_{0.2}$Ir$_{0.2}$Ru$_{0.2}$Rh$_{0.2}$ core-shell nanocrystals during the 150th cycle. The band species assignments for the colored boxes in panel (a) are consistent with those shown in Figure 4 and listed in Table S6.

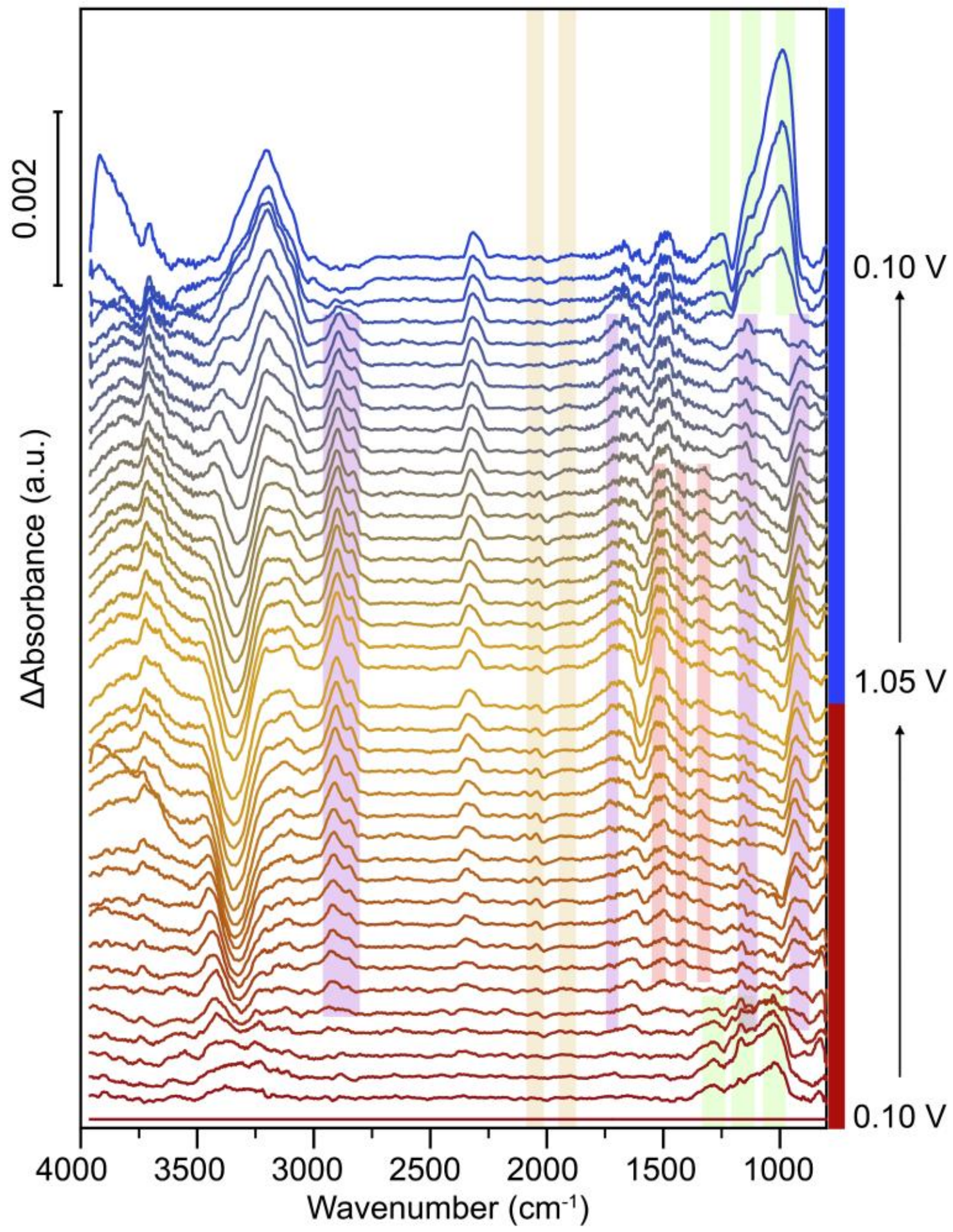

Figure S21. In situ SEIRAS spectral analysis during the first EOR cycle for Pd nanocubes in 1 M KOH + 1 M ethanol (Ar-saturated) electrolyte with a potential step of 0.05 V at a potential window of 0.1-1.05 V vs. RHE. The bands highlighted in the green-shaded region (1030, 1175, and 1300 cm^{-1}) correspond to strong ethanol adsorption. The purple-shaded region, characterized by representative peaks at 1730 cm^{-1} (C=O stretching) and 2700-2950 cm^{-1} (C-H stretching), is attributed to the formation of acetaldehyde. The red-shaded region, with features at 1520 cm^{-1} (antisymmetric O-C-O stretch), 1410 cm^{-1} (symmetric O-C-O stretch), and 1335 cm^{-1} (in-plane C-H bending), indicates the formation of acetate species. Additionally, the bands at 1910 and 2040 cm^{-1} are assigned to linearly and multiply bound CO_{ad} species, respectively [33, 38, 65].

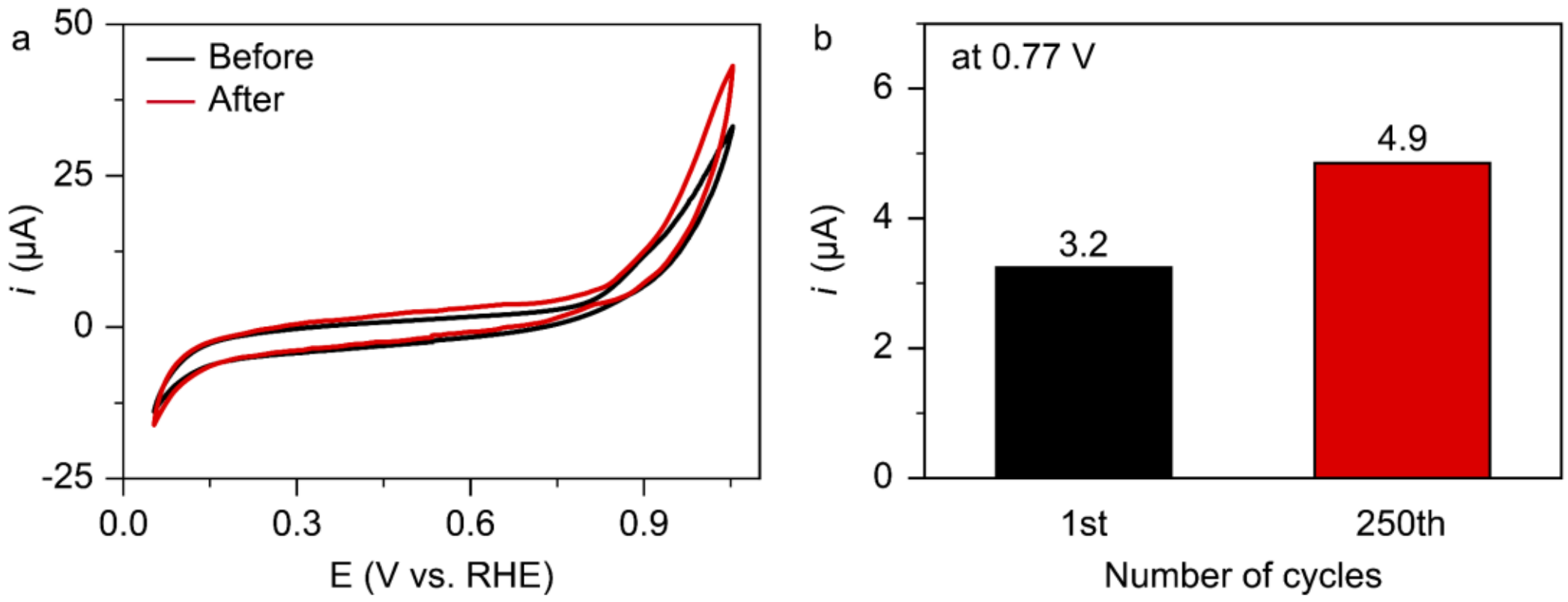

Figure S22. Electrocatalytic Measurements in Ar-saturated 1M KOH + 1M CH_3CHO for $Pd@Pd_{0.2}Pt_{0.2}Ir_{0.2}Ru_{0.2}Rh_{0.2}$ core-shell nanocrystals pre- and post-EOR (250 cycles). (a) CV profiles. (b) Current at 0.77 V vs. RHE.

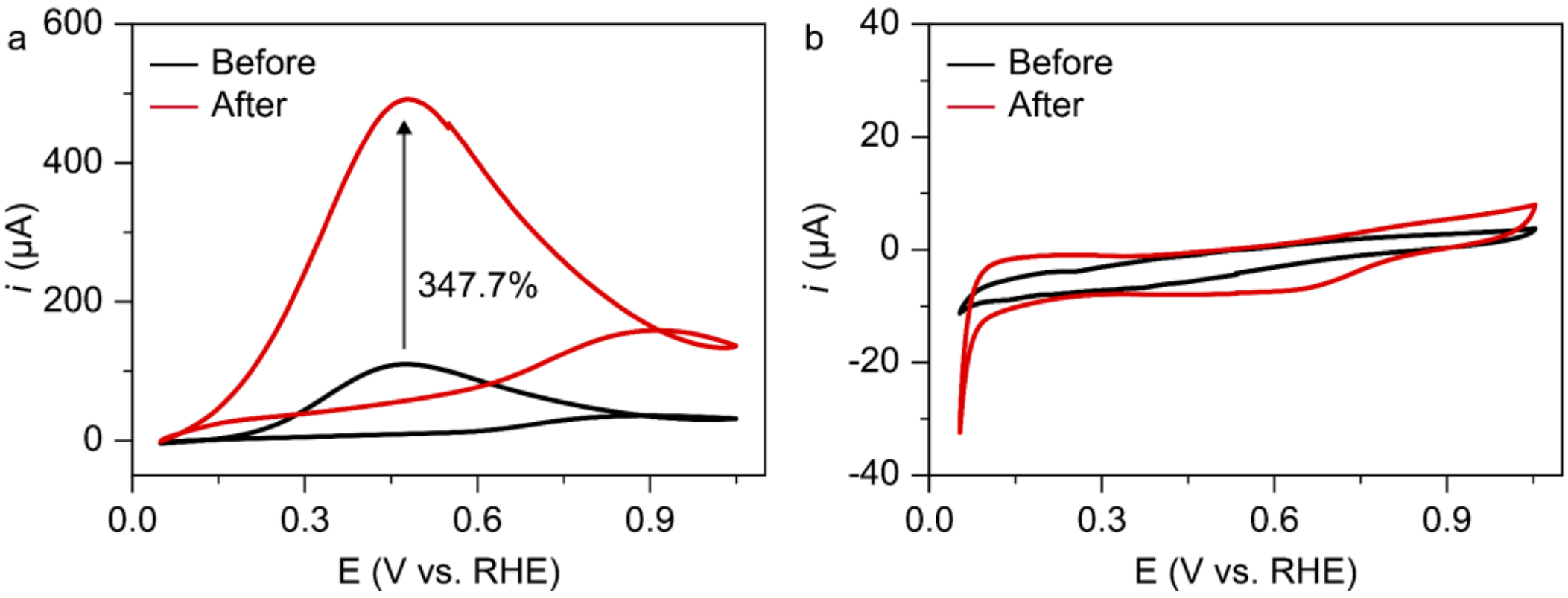

Figure S23. CV profiles in (a) Ar-saturated 1M KOH + 1M HCOOH, and (b) Ar-saturated 1M KOH + 1M CH_3COOH for Pd@$Pd_{0.2}Pt_{0.2}Ir_{0.2}Ru_{0.2}Rh_{0.2}$ core-shell nanocrystals pre- and post-EOR (250 cycles).

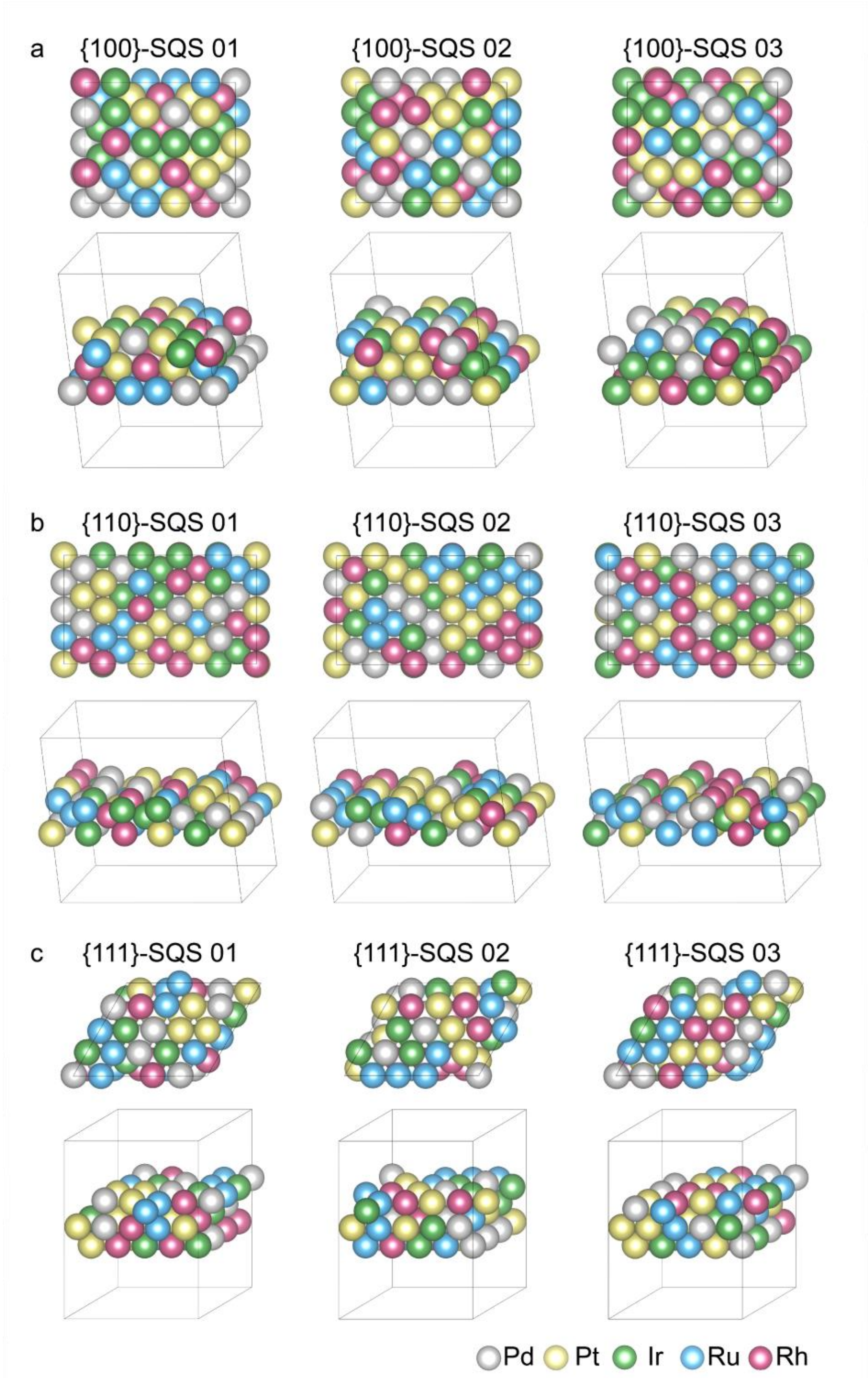

Figure S24. Top and side views of $Pd_{0.2}Pt_{0.2}Ir_{0.2}Ru_{0.2}Rh_{0.2}$ HEA slab models with different exposed facets. (a) {100}, (b) {110}, and (c) {111}. For each facet, three representative special SQS models, labeled SQS 01 to SQS 03, were generated to represent the random atomic distribution in the quinary HEA system. These models were subsequently used for high-throughput sampling of atomic $HCOO_{ad}$ adsorption configurations on the HEA facets. Black lines denote the unit-cell boundaries.

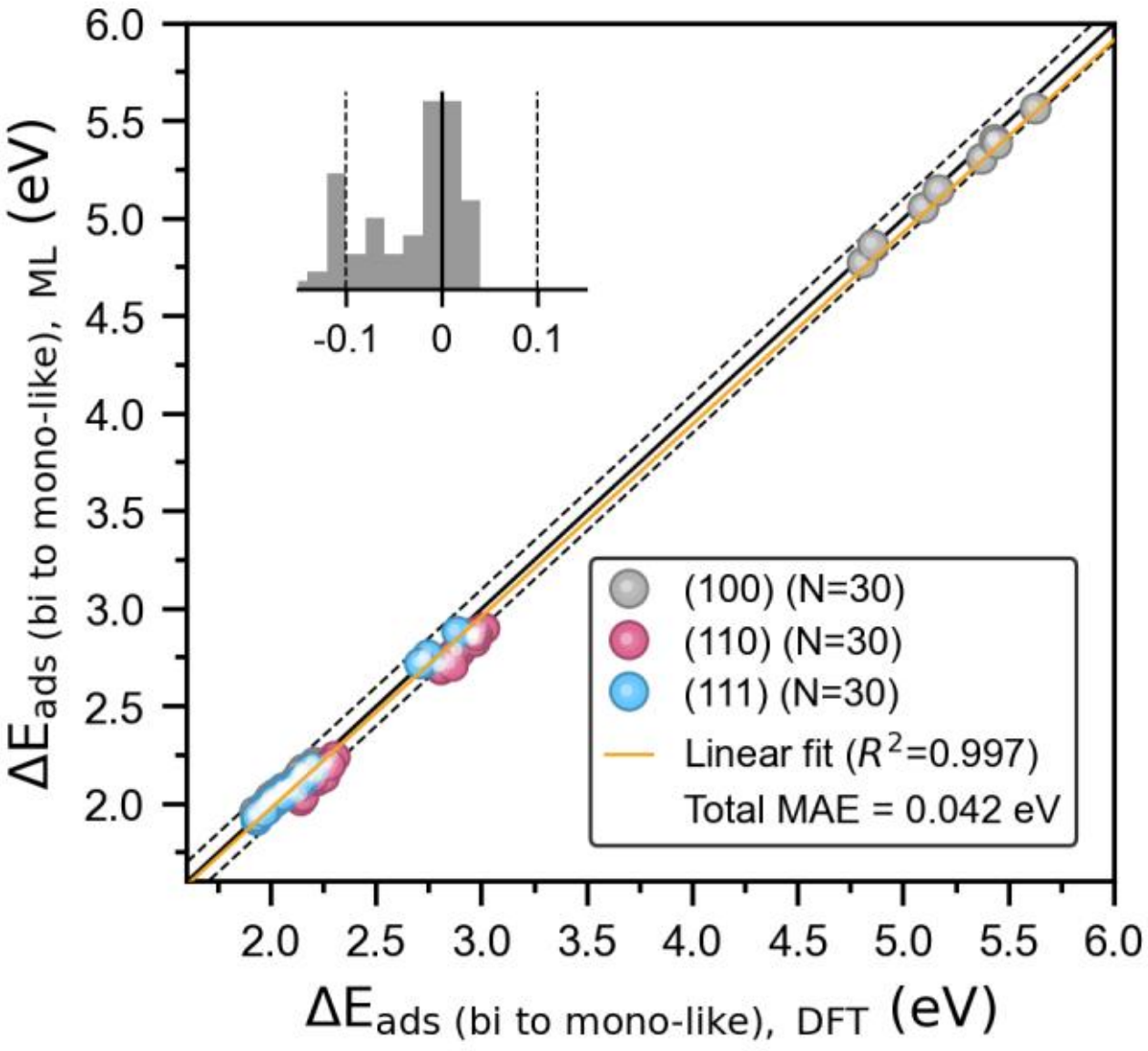

Figure S25. Parity plot comparing the adsorption energy difference, $\Delta E_{ads\ (bi\text{-}HCOO\ to\ mono\text{-}like)}$, calculated by the UMA (uma-m-1p1) machine-learning interatomic potential model with the corresponding DFT reference values for $Pd_{0.2}Pt_{0.2}Ir_{0.2}Ru_{0.2}Rh_{0.2}$ HEA slabs with different exposed facets. A total of 90 adsorption sites were evaluated, including 15 bidentate sites × 2 mono-like configurations on M1 or M2 × 3 facets. The solid black line represents ideal agreement, while the dashed lines indicate a ±0.10 eV deviation range. The linear fit shows an R^2 value of 0.997, and the overall mean absolute error (MAE) is below 0.05 eV, validating that the ML model accurately reproduces the DFT-calculated adsorption energies.

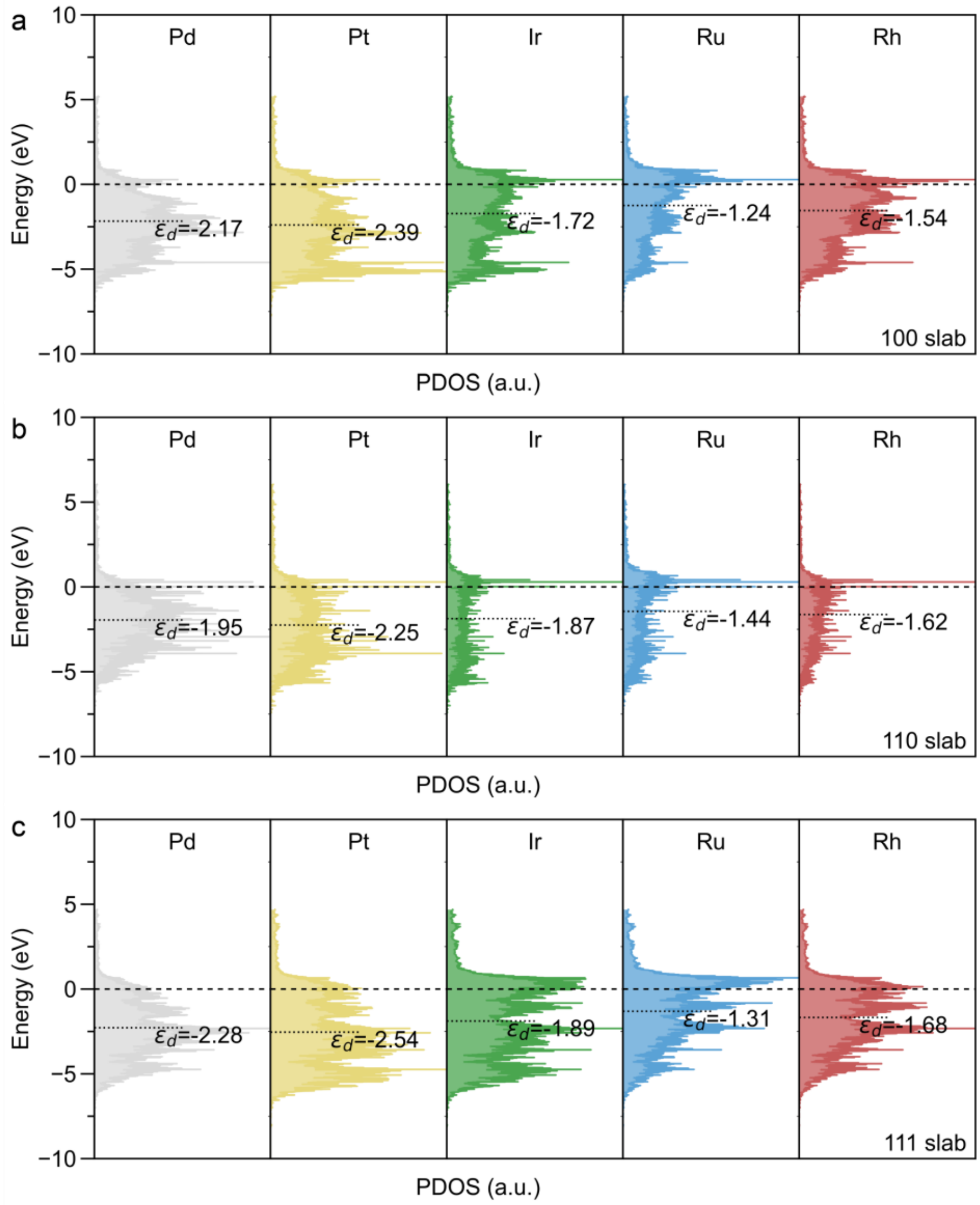

Figure S26. PDOS of Pd, Pt, Ir, Ru, and Rh in $Pd_{0.2}Pt_{0.2}Ir_{0.2}Ru_{0.2}Rh_{0.2}$ HEA slabs with different exposed facets. (a) HEA {100} slab, (b) HEA {110} slab, and (c) HEA {111} slab.

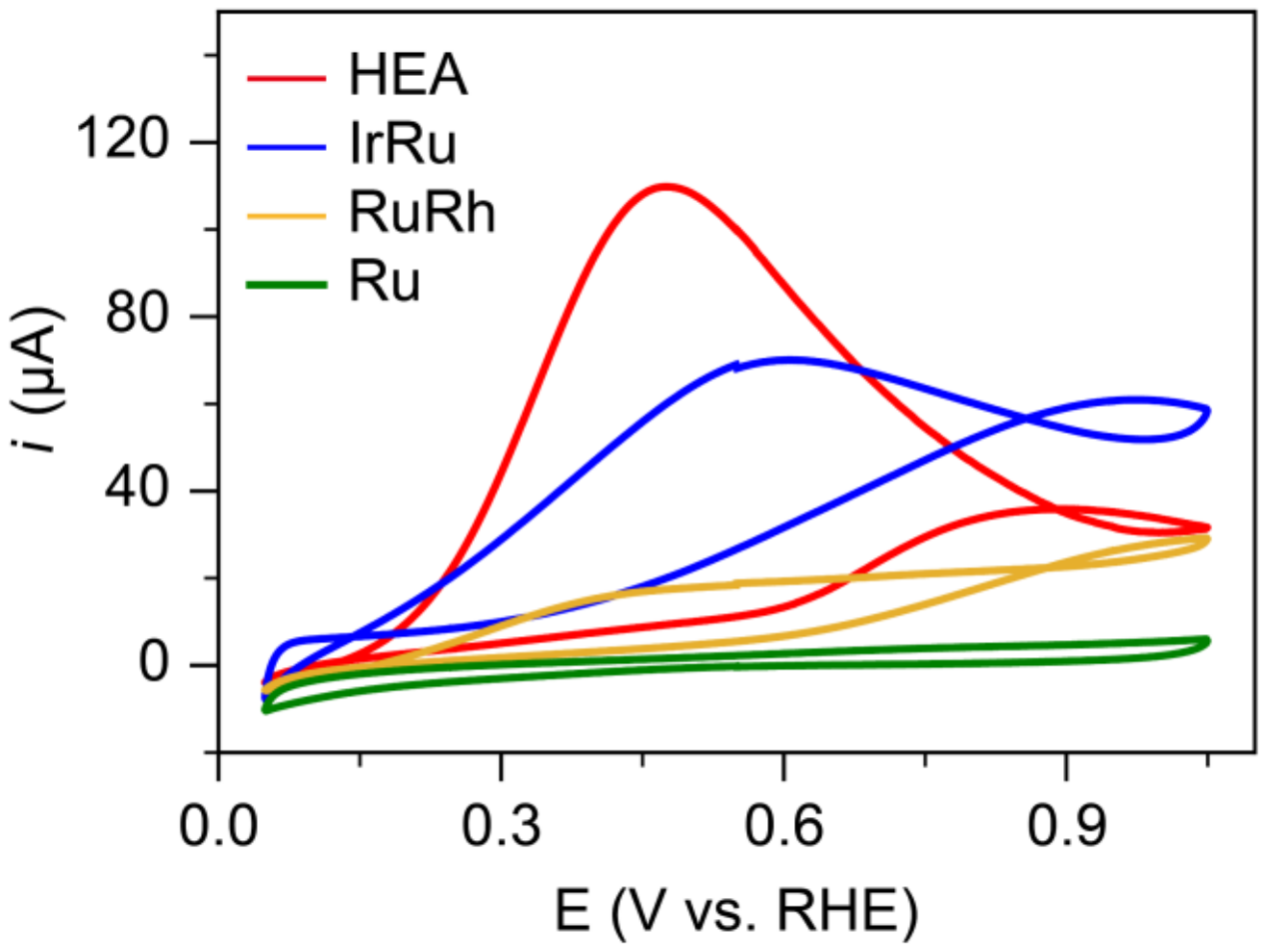

Figure S27. CV profiles during the first cycle in Ar-saturated 1 M KOH + 1 M HCOOH for single-metal, binary-alloy, and HEA core-shell nanocubes, including Pd@Ru, Pd@$Ru_{0.5}Rh_{0.5}$, and Pd@$Ir_{0.5}Ru_{0.5}$, and Pd@$Pd_{0.2}Pt_{0.2}Ir_{0.2}Ru_{0.2}Rh_{0.2}$. Compared with these Ru-containing controls, the Pd@$Pd_{0.2}Pt_{0.2}Ir_{0.2}Ru_{0.2}Rh_{0.2}$ HEA catalyst exhibits the highest peak current and the lowest peak potential (109.8 μA at 0.48 V), substantially outperforming Pd@$Ir_{0.5}Ru_{0.5}$ (70.0 μA at 0.61 V), Pd@$Ru_{0.5}Rh_{0.5}$ (29.2 μA at 1.05 V), and Pd@Ru (6.03 μA at 1.05 V). These results indicate that the strongly binding Ru-based motifs alone do not deliver efficient oxidation/removal of formate-related species; instead, the best performance emerges only when these motifs are embedded in a multicomponent HEA environment that weakens over-stabilization and provides more favorable neighboring adsorption/oxidation geometries.

Tables

Table S1. Representative examples of noble metal-based electrocatalysts for EOR in alkaline media.

Composition	Electrolyte	Onset Potential (V vs. RHE)	Peak Potential (V vs. RHE)	j_f/j_b ratio	C1 pathway selectivity	Stability test method	Ref.
PtPdRhRuCoNi	1 M KOH + 1 M EtOH	0.38	0.896	1.83	N/A	4000 s loss 93.4%	*Chem. Commun.* **2026**, 62, 4071-4075
Pd-$(CuMnFeNiCo)_3O_4$,/C	1 M KOH + 1 M EtOH	0.41	0.77	1.09	N/A	500 cycles loss 10%	*J. Solid State Electrochem.* **2026**, 30, 89-105
PdSn NWs	1 M KOH + 1 M EtOH	0.50	0.85	0.77	N/A	500 cycles loss 77.1%	*Inorg. Chem. Front.* **2025**, 12, 7261-7270
PdSnFeCoNi/c-PdSn	1 M KOH + 1 M EtOH	0.453	0.79	1.04	64.1%	2000 cycles loss 9.4%	*Nat. Commun.* **2025**, 16, 3403
PdRhFeCoMo metallene	1 M KOH + 1 M EtOH	0.152	0.654	0.74	84.12%	4000 cycles loss 28.96%	*Adv. Sci.* **2024**, 11, 2409109
PtPdFeCoNiSnMn HEA (PdPt HEA)	1 M KOH + 1 M EtOH	0.255	0.77	~1.40	>92%	50000 cycles loss 0.3%	*Joule* **2023**, 7, 587-602
Pd/Co@N-C	1 M KOH + 1 M EtOH	~0.42	~1.0	~1.17	>80%	500 cycles loss 15.8%	*Nat Commun.* **2023**, 14, 1346
a-PdCu-6.5 nm	1 M KOH +1 M EtOH	0.48	0.79	1	69.6%	1000 cycles loss 33.7%	*Nano Lett.* **2022**, 22, 7028
FeCoNiSn@FeCoNiSnPd	1 M KOH +1 M EtOH	0.37	0.78	0.96	N/A	2000 cycles loss 8.2%	*Adv. Funct. Mater.* **2022**, 32, 2204643
Pd NPs@Ni SAC	1 M KOH +1 M EtOH	0.3	0.91	1.08	28%	4000 cycles loss 10%	*J. Mater. Chem. A* **2022**, 10, 6129
PdZn/NC@ZnO	1 M KOH +1 M EtOH	0.50	0.82	1.38	N/A	Negligible loss after 2000 cycles	*Nat. Commun.* **2021**, 12, 5273
Pt_3Ag alloy wavy NWs	1 M KOH +1 M EtOH	0.30	0.81	0.93	N/A	6000 s loss 97.5%	*Nano Res.* ***2020***, 13, 1472
PdAu Aerogel	1 M KOH +1 M EtOH	0.42	0.91	0.71	N/A	2000 s loss 78%	*Angew. Chem.* **2020**, 132, 5755
SANi-Pt NWs	1 M KOH +1 M EtOH	0.30	0.785	1.92	N/A	N/A	*Nat. Catal.* ***2019***, 2, 495
Core-shell $Pt_{56}Cu_{28}Ni_{16}$ tetrahedra	1 M KOH +1 M EtOH	0.22	0.79	1.32	N/A	3600 s loss 47%	*Nano Lett.* ***2019***, 19, 5431
Core@shell Au@PtIr	1 M KOH +1 M EtOH	0.35	0.90	N/A	57%	N/A	*J. Am. Chem. Soc.* **2019**, 141, 9629
Core@skin PtBi@Pt	1 M KOH +1 M EtOH	0.52	0.82	1.59	*Above 90%*	*5000 cycles loss 21.4%*	*J. Mater. Chem. A* **2019**, 7, 5214
$Pt_{54}Rh_4Cu_{42}$ CNBs	1 M KOH +1 M EtOH	0.36	0.78	*2.1*	*40.7%*	*1000 s loss 17.2%*	*Adv. Energy Mater.*

							**2018**, 8, 1801326
Dealloyed $PtPd_3Ag_5/C$-	1 M KOH +1 M EtOH	0.37	0.87	*1.62*	*NA*	*1800 s loss ~57%*	*Electrochim. Acta* **2017**, 236, 72
$Pt_{68}Cu_{32}$ nanoalloys	0.5 M KOH +0.5 EtOH	0.37	0.97	*1.89*	*NA*	*3600 s loss ~94%*	*Nanoscale* **2017**, 9, 2963
PtMo/C	0.5 M KOH +0.5 EtOH	0.31	0.86	*1.62*	*Mainly C2*	*NA*	*Appl. Catal., B* **2017**, 203, 654
$Pd/Ni(OH)_2/RGO$	1 M KOH +1 M EtOH	0.37	0.93	*1.05*	*26%*	*40000 cycle loss <10%*	*Adv. Mater.* **2017**, 29, 1703057
Pd Aerogel	1 M NaOH +1 M EtOH	0.32	0.82	*~1.45*	*NA*	*200 cycles loss 8.8%*	*Mater. Chem. A* **2017**, 5, 10244
PtCo-NWs/CF	0.1 M KOH +0.5 M EtOH	0.37	0.87	*1.51*	*NA*	*NA*	*Electrochim. Acta* ***2016***, 199, 218
Pd/Carbon Black	1 M NaOH +1 M EtOH	0.37	0.91	*~1.26*	*NA*	*NA*	*Int. J. Hydrogen Energy* **2015**, 40, 12382
PtPd (1:3)/RGO/GC	1 M KOH +1 M EtOH	0.28	0.78	*~1.2*	*NA*	*100 cycles loss 6%*	*ACS Appl. Mater. Interfaces* **2014**, 6, 3607
$Pd@Pd_{0.2}Pt_{0.2}Ir_{0.2}Ru_{0.2}Rh_{0.2}$-core-shell severely beveled cubes (250th EOR cycles)	1 M KOH +1 M EtOH	0.332	0.768	2.01	Over 73%, with negligible C2 product detected	(1) Increased by 954% over 1-250 EOR cycles (2) 45 000 s at 0.5 V, with negligible change between the pre- and post-CV profiles	This work

Table S2. ICP-OES results of the atomic compositions in the $Pd@Pd_{0.2}Pt_{0.2}Ir_{0.2}Ru_{0.2}Rh_{0.2}$ core-shell nanocrystals.

Sample name	Composition (at %)				
	Pt	Pd	Ir	Ru	Rh
After 0 EOR cycle (pristine sample)	81.5	4.8	4.8	4.4	4.5
After 50 EOR cycles	82.4	4.4	4.9	4.1	4.2
After 150 EOR cycles	82.1	4.4	5	4.2	4.2
After 250 EOR cycles	82.2	4.4	4.9	4.3	4.2
After 500 EOR cycles	82.4	4.4	5	4.1	4.2
Pristine sample before 45000 s CA test	84.3	3.2	4.0	4.3	4.1
After 250 EOR cycles + 45000 s CA test	85.4	3.1	3.6	4.1	3.8

Table S3. Summary of XPS fitting analysis for $Pd@Pd_{0.2}Pt_{0.2}Ir_{0.2}Ru_{0.2}Rh_{0.2}$ core-shell nanocrystals before and after 250 EOR cycles and following the long-term CA test for 45,000 s. Common chemical states were assigned according to reference standards.

**N. of Cycles**	**Pd^0/Pd^{2+} $3d_{5/2}$**	**Pt^0/Pt^{2+} $4f_{7/2}$**	**Ir^0/Ir^{4+} $4f_{7/2}$**	**Ru^0/Ru^{4+} $3p_{3/2}$**	**Rh^0/Rh^{3+} $3d_{5/2}$**
LG% [a]	50%/20%	50%/20%	70%/20%	60%/20%	60%/35%
Pre-EOR (position; eV)	335.7/336.7	71.6/72.9	60.9/61.9	461.8/465.2	307.5/308.5
Pre-EOR (area; %)	91.2/8.8	95.9/4.1	89.4/10.6	88.0/12.0	80.9/19.1
Post-EOR (position; eV)	335.9/336.9	71.8/73.1	61.1/62.1	461.9/465.3	307.6/308.6
Post-EOR (area; %)	87.9/12.1	96.8/3.2	88.3/11.7	84.8/15.2	79.3/20.7
After 45000 s CA test (position; eV)	335.6/336.6	71.6/72.9	60.9/61.9	462.0/465.4	307.6/308.6
After 45000 s CA test (area; %)	87.9/12.1	93.7/6.3	85.6/14.4	87.0/13.0	87.5/12.5
Average state (Pre/post)	+0.18/+0.24	+0.08/+0.06	+0.42/+0.47	+0.48/+0.61	+0.57/+0.62
Average state (After CA)	+0.24	+0.12	+0.57	+0.52	+0.38

[a] Lorentzian/Gaussian function fitting ratio.

Table S4. Structural parameters derived from EXAFS fitting analyses of $Pd@Pd_{0.2}Pt_{0.2}Ir_{0.2}Ru_{0.2}Rh_{0.2}$ core-shell nanocrystals after 250 cycles of EOR.

	Path	CN	R (Å)	σ^2 (Å^2)	*R-factor*[a]	S_0^2 [b]	ΔE [c](eV)
Pd_Foil	Pd-Pd	12.00	2.74	0.005	0.007	0.70	-0.215
Pd_nanocubes [d]	Pd-Pd	7.14	2.74	0.004	--	0.81	--
Pd_Pd@HEA	Pd-4d	7.82	2.78	0.003	0.007	0.70	-0.215
	Pd-5d	1.00	2.81	0.007	0.007	0.70	-0.215
	Pd-O	0.53	1.94	0.005	0.007	0.70	
Pt_Foil	Pt-Pt	12.00	2.76	0.004	0.031	0.75	7.677
HEA_Pt	Pt-4d	5.30	2.72	0.004	0.031	0.75	7.677
	Pt-5d	4.22	2.73	0.003	0.031	0.75	7.677
	Pt-O	1.11	1.99	0.012	0.031	0.75	7.677
Ir_Foil	Ir-Ir	12.00	2.71	0.003	0.023	0.76	8.000
HEA_Ir	Ir-4d	4.04	2.71	0.003	0.023	0.76	8.000
	Ir-5d	2.67	2.71	0.004	0.023	0.76	8.000
	Ir-O	1.81	1.94	0.007	0.023	0.76	8.000
Ru_Foil	Ru-Ru	12.00	2.67	0.003	0.040	0.69	0.139
HEA_Ru	Ru-4d	4.42	2.72	0.008	0.040	0.69	0.139
	Ru-5d	2.47	2.70	0.006	0.040	0.69	0.139
	Ru-O	2.75	1.98	0.003	0.040	0.69	0.139
Rh_Foil	Rh-Rh	12.00	2.68	0.003	0.020	0.68	-2.24
HEA_Rh	Rh-4d	4.67	2.67	0.004	0.020	0.68	-2.24
	Rh-5d	5.00	2.69	0.003	0.020	0.68	-2.24
	Rh-O	1.12	1.96	0.003	0.020	0.68	-2.24

[a] *R-factor* = $\sum(\text{data-fit})^2/\sum \text{data}^2)$

[b] S_0^2 : fixed amplitude reduction factor based on first-shell fitting of metal foils

[c] ΔE : energy shift.

[d] The fitting data for Pd nanocubes reference was derived from our previous work for comparison [18].

Table S5. ICP-OES results of the five PGMs in the electrolyte after EOR cycling and CA testing for Pd@$Pd_{0.2}Pt_{0.2}Ir_{0.2}Ru_{0.2}Rh_{0.2}$ core-shell nanocrystals. In both cases, the concentrations of each element in the electrolyte were below 0.005 ppm (5 ppb) and fluctuated around zero, indicating negligible metal dissolution under the applied experimental conditions. The apparent negative concentrations obtained by ICP-OES are artifacts of blank correction and calibration intercept uncertainty at ultralow analyte levels. Since the measured signals are indistinguishable from the blank/background, these values are interpreted as below the detection/quantification limit rather than as real negative concentrations.

Sample name	Concentration (ppm)				
	Pt	Pd	Ir	Ru	Rh
Electrolyte after 250 EOR cycles	0.001±0.005	-0.003±0.008	0.005±0.003	0.002±0.003	0.003±0.008
Electrolyte after 250 EOR cycle + 45000 s CA test	0.002±0.004	-0.003±0.006	0.002±0.001	0.000±0.004	0.001±0.009

Table S6. SEIRAS band Assignments for EOR.

Wavenumber (cm^{-1})	Assignment	Bonding Type	Potential Dependence
880	Carbonate formation	υ(CO_3^{2-}) symmetric stretching	Starts from 0.4 V
1430	Carbonate formation	υ(CO_3^{2-}) asymmetric stretching	Starts from 0.4 V
2332	CO_2 evolution	υ(CO_2) stretching	Increasing with potential (starts from 0.3 V)
1040	CH_3CH_2OH*,CH_3CHO*, CH_xCO* intermediates	υ(C-O) stretching	Increasing with potential
1154	CH_3CH_2OH*,CH_3CHO*, CH_xCO* intermediates	υ(C-C) / υ(C-O) stretching	Increasing with potential
1513	CH_3CH_2OH*,CH_3CHO*, CH_xCO* intermediates	δ(CH_3) bending or H-bonded OH	Increasing with potential
1673	CH_3CH_2OH*,CH_3CHO*, CH_xCO* intermediates	υ(C=O) acetaldehyde-like or adsorbed carbonyl	Increasing with potential
2770-3010	C-H stretch of ethoxy/acetyl intermediates (forward scan)	υ(C-H) stretching	Increasing with potential
960	CH_3COO*, HCOO* intermediates formation	C-C or C-O stretching	Occur in backward scan
1275	CH_3COO*, HCOO* intermediates formation	υ(COO*) symmetric stretching or δ(CH_3) bending	Occur in backward scan
1570	CH_3COO*, HCOO* intermediates formation	υ(COO*) asymmetric carboxylate	Occur in backward scan
2820, 2900	CH_3COO*, HCOO* intermediates formation	υ(C-H) stretching or υ(CH_3) stretching	Occur in backward scan
3725, 3840	Fresh OH* adsorbing on bare metal	υ(O-H) stretching (M-OH*)	During CO_2 formation
3680, 3740	Hydrogen-bonded OH* or weak OH* on e^- rich sites	υ(O-H) stretching (COO*-OH*)	During COO* (HCOO*, CH_3COO*) formation
3062-3418	water	υ(O-H) stretching (broad)	Broad band increasing
